# Delineating precise surgical resection areas: recent advances in NIR-II fluorescence imaging molecular probes

**DOI:** 10.3389/fonc.2026.1872947

**Published:** 2026-09-21

**Authors:** Di Liu, Fanwei Zeng, Lipeng Sun, Shuai Zhang, Tingting Ren, Jie Zang, Xiaodong Tang

**Affiliations:** 1 Musculoskeletal Tumor Center, Peking University People’s Hospital, Beijing, China; 2 Graduate School, Hebei Medical University, Shijiazhuang, Hebei, China

**Keywords:** intraoperative guidance, molecular probes, near-infrared II fluorescence imaging, precision surgery, R0 resection, surgical navigation, targeted imaging agents, tumor margin delineation

## Abstract

Precise delineation of surgical resection margins is a pivotal determinant of oncologic surgery outcomes, with R0 resection serving as the gold standard for maximizing local control and long-term survival. However, intraoperative identification of tumor boundaries remains challenging. Conventional tools such as frozen section pathology offer limited field coverage, are labor-intensive, time-consuming, and may yield sampling errors, particularly in anatomically complex or highly infiltrative lesions. Near-infrared II (NIR-II, 1000–1700 nm) fluorescence imaging has emerged as a transformative modality for real-time intraoperative navigation. Leveraging reduced photon scattering, minimal tissue autofluorescence, and deeper penetration compared with NIR-I and other optical approaches, NIR-II imaging enables high-contrast visualization of tumor margins while preserving adjacent normal structures. Over the past decade, the molecular probe landscape for NIR-II imaging has rapidly evolved—from early non-specific small molecules and inorganic nanomaterials to the latest generation of targeted agents designed to bind tumor-specific biomarkers. These advances have facilitated more accurate tumor–normal tissue contrast in a range of surgical contexts, including glioma, hepatobiliary malignancies, colorectal cancer, and head–neck tumors. Several first-in-human and ongoing clinical trials have demonstrated the feasibility, safety, and intraoperative utility of NIR-II molecular probes. Nevertheless, issues related to regulatory approval, probe pharmacokinetics, real-time quantitative imaging, and integration with multimodal surgical systems remain to be addressed. This review summarizes current concepts of precise surgical resection margins, outlines the origin and principles of NIR-II fluorescence imaging, and provides an in-depth discussion of targeted molecular probe development, preclinical and clinical progress, and their role in tumor margin delineation. Finally, we highlight existing limitations and future opportunities for translating NIR-II probes into routine surgical oncology practice.

## 1 Introduction

The precision of surgical resection aims to achieve complete removal of cancerous tissue with clear margins while preserving healthy tissue, thereby accomplishing an R0 resection. Positive or close positive margins (R1/R2) are associated with higher recurrence rates and poorer patient outcomes, making the attainment of R0 resection critically important (1, 2). However, even with meticulous surgical techniques, up to 30–40% of cancer resections still result in positive or close positive margins, highlighting the intraoperative challenge of distinguishing tumor tissue from normal tissue (3, 4). This issue is further complicated by subclinical tumor spread and the limitations of human vision in detecting microscopic lesions (4, 5).

We argue that intraoperative assessment is essential for guiding immediate surgical decisions. In pathology-assisted settings, this entails evaluating fresh biopsies or resection margins. Building on this, Voskuil et al. introduced intraoperative pathology-assisted surgery (IPAS), which we consider a valuable paradigm leveraging imaging to facilitate rapid surgeon-pathologist collaboration and guide further resection (6). Traditionally, surgeons rely on visual inspection and palpation to assess tumor boundaries, which is inherently subjective and can miss hidden tumor areas (7). Intraoperative frozen section pathology is the gold standard for checking margins (8), but it has significant drawbacks. It’s time-consuming, disrupts the flow of surgery, and only samples a small part of the specimen, potentially missing tumor in unsampled areas (9). Preparing and interpreting frozen slides can take 20–30 minutes or more, which can extend anesthesia time (10). Other techniques including intraoperative ultrasound or MRI can aid in localizing larger tumors but lack the resolution required to detect microscopic disease, and their continuous intraoperative application is often impractical (11, 12). These limitations make it difficult for surgeons to accurately delineate tumor boundaries in real time, balancing the need for a complete resection with the desire to avoid unnecessary removal of healthy tissue.

Intraoperative imaging techniques like radiologic guidance or endoscopic inspection can be useful for certain tumors (13). While preoperative MRI, CT, or PET scans help plan the surgery, they aren’t easily adaptable for real-time use during the procedure (14). Some newer tools, such as intraoperative MRI suites or ultrasound probes, offer anatomical guidance but still struggle to detect microscopic disease and are often not available during resection itself (15). As a result, surgeons often face a difficult decision: removing extra tissue for safety, which can lead to more complications, or risking leaving cancerous tissue behind in an effort to preserve function.

Fluorescence-guided surgery (FGS) represents a promising solution, as it enables real-time labeling of malignant tissue using molecular probes, thereby providing clear visual contrast between tumors and healthy tissue and assisting surgeons in achieving more precise intraoperative decision-making (16, 17). As shown in **Figure 1**, the process can be dynamically observed in real time via a display screen. FGS has already shown success in certain applications—such as brain tumor surgery, where the use of 5-aminolevulinic acid (5-ALA) to induce fluorescence in high-grade gliomas has increased complete resection rates and improved patient outcomes (18). Together with extensive preclinical evidence, these clinical successes have contributed to the initiation of more than 20 clinical trials of fluorescence-guided surgery in oncology by the mid-2010s (19). The goal of FGS is to create a “precision surgical area” by mapping out the true boundaries of the tumor, including its microscopic extensions, so surgeons can remove just the right amount of tissue—ensuring complete tumor removal while avoiding damage to healthy organs or critical structures.

**Figure 1:**
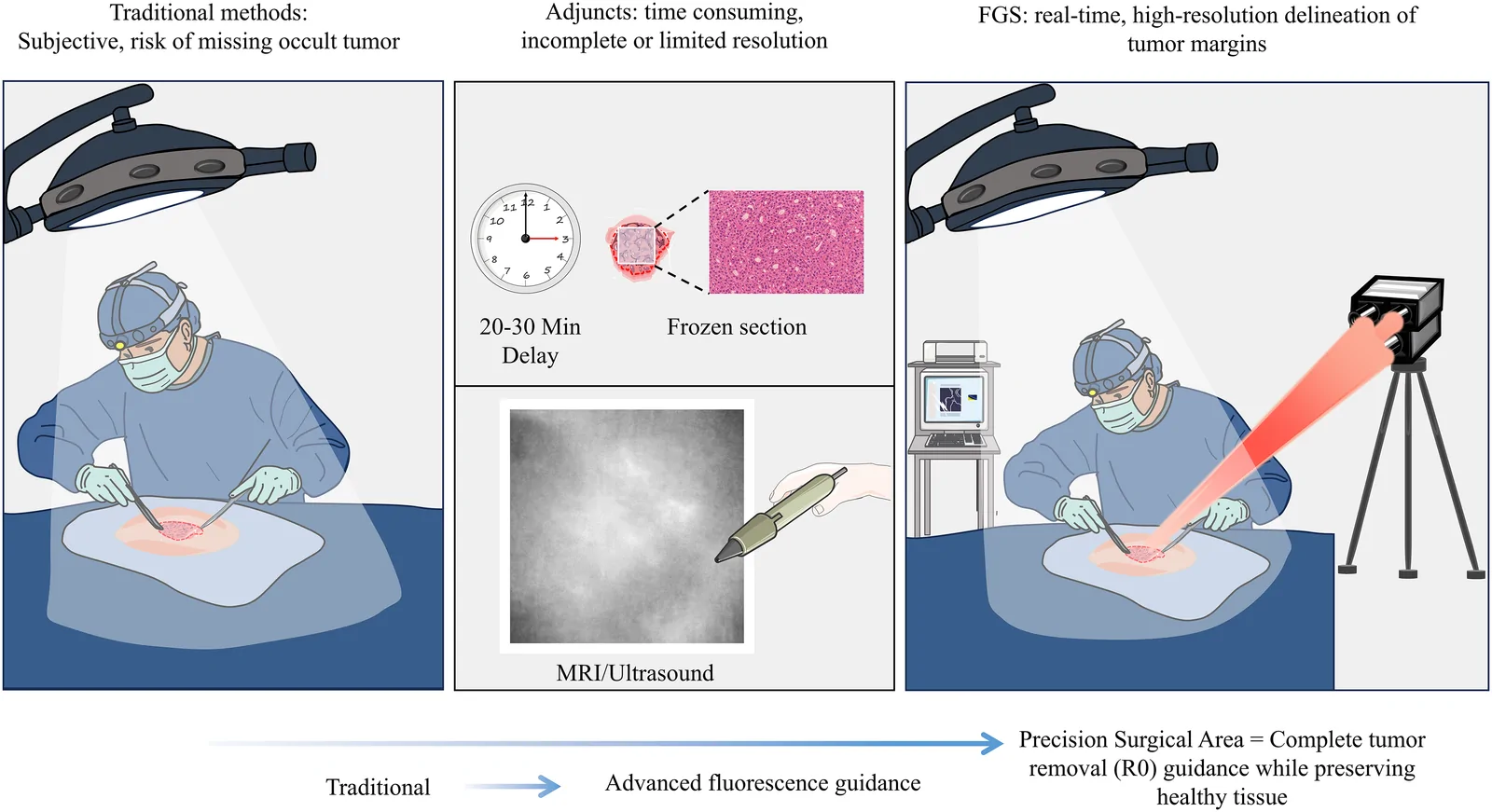
Schematic diagram of precision surgery evolution limitations of traditional surgery. Surgeons rely on visual inspection and palpation for tumor resection. Tumor margins are often indistinct, with subclinical infiltrating tissue blending into surrounding healthy tissue. Current Auxiliary Technologies: Techniques such as intraoperative frozen section pathology, intraoperative MRI, or ultrasound are available. However, these methods suffer from drawbacks including prolonged duration, incomplete information, and limited resolution. Emerging Solution, FGS: Under laser excitation within the intraoperative field of view, tumors are visualized in real-time on screens, clearly delineating their location and margins from normal tissue. This allows for precise manipulation of surgical instruments, achieving an R0 resection margin.

A summary of current methods for intraoperative margin assessment, along with their respective advantages and limitations, is provided in **Table 1**. Given the inherent limitations of existing techniques, there is an urgent clinical need for real-time, highly sensitive, and specific modalities to guide surgical resection. Near-infrared fluorescence imaging has emerged as a highly promising intraoperative imaging technology and is receiving growing attention. Within this field, NIR-II fluorescence imaging is currently a major research focus. Preclinical studies suggest that, compared with NIR-I, NIR-II offers the potential for improved signal-to-background ratios and reduced tissue scattering; however, its clinical superiority requires further validation. In practice, current clinical imaging platforms remain primarily optimized for indocyanine green (ICG)-based NIR-I imaging. Constrained by hardware limitations such as excitation/emission filter design and detector optimization, as well as the limited number of clinically approved tumor-targeted probes, these platforms still exhibit restricted sensitivity for most tumor-specific fluorophores beyond ICG.

**Table 1:** Common techniques for intraoperative tumor margin assessment and their limitations.

Method	Principle	Advantages	Limitations
Surgeon’s visual inspection and palpation	Macroscopic observation of color/texture; tactile feedback for firmness	Immediate and always available; uses surgeon’s experience	Subjective; cannot detect microscopic disease or deep margins; subtle tumor edges can be missed (20)
Frozen section pathology	Rapid histological examination of resected margin slices (frozen tissue sections)	Gold standard for identifying microscopic tumor at margins; informs immediate re-excision if positive	Time-consuming (each analysis may take 20–30 min); samples only a fraction of the margin (risk of sampling error); requires pathology support; tissue freezing artifacts can affect accuracy (21)
Intraoperative ultrasound or radiologic imaging	Real-time imaging (ultrasound, CT, etc.) for gross tumor visualization during surgery	Identifies deeper structures; ultrasound can guide resection of non-palpable tumors (e.g., liver lesions) (22)	Limited resolution – cannot detect microscopic residuals; not easily adaptable for continuous use during resection; may require special equipment and training (23)
FGS using molecular probes (NIR fluorescence)	Tumor-specific or tumor-selective fluorophores accumulate in cancer and emit light under NIR excitation, highlighting tumor tissue in real time in the surgical field	Real-time, high sensitivity detection of tumor deposits; wide field of view; integrates with surgeon’s vision; can detect sub-millimeter disease (24)	Only a few probes are clinically approved (e.g., ICG for perfusion, not tumor-specific) (25); depth of light penetration can be limited (especially in visible/NIR-I range) (26); requires specialized imaging cameras; potential for false positives if probe lacks specificity

## 2 NIR-II fluorescence imaging: origin, principles, and advantages

### 2.1 The second near-infrared window: definition and principles

NIR fluorescence imaging uses special dyes that emit light in the near-infrared spectrum to visualize biological tissues. The NIR range is divided into two parts: the first NIR window (NIR-I), which covers about 700–900 nm, and the second NIR window (NIR-II), which spans approximately 1000–1700 nm (also known as shortwave infrared, or SWIR) (27). As illustrated in **Figure 2**, compared to visible light (400–700 nm), fluorescence imaging in the NIR region offers distinct advantages. Light with longer wavelengths undergoes reduced absorption and scattering by tissues, which not only enables greater penetration depth but also minimizes signal attenuation (28). Additionally, tissue autofluorescence is much lower in the NIR range, especially in NIR-II, resulting in clearer images with better contrast (28).

**Figure 2:**
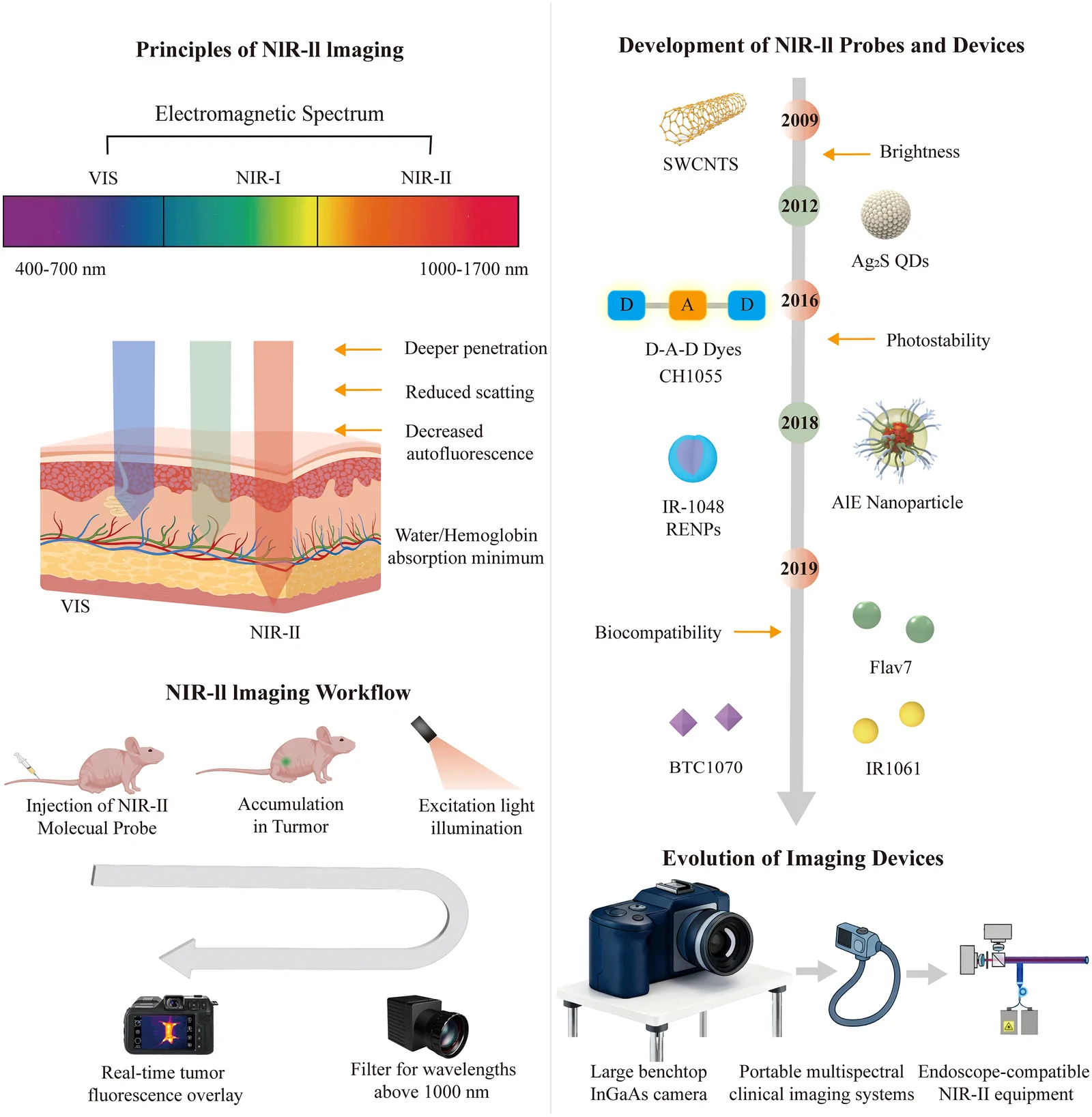
Principles and advancements of NIR-II fluorescence imaging. The NIR-II spectrum (1000–1700 nm) features reduced scattering, increased penetration depth, and lowered autofluorescence. Workflow: Following intravenous injection, NIR-II probes accumulate in tumors and are visualized using an NIR-II camera (>1000 nm filter) under excitation, enabling real-time fluorescence overlay. Timeline (2009-present) highlights the emergence of various probe categories—including single-walled carbon nanotubes, donor–acceptor–donor (D-A-D) dyes, Ag_2_S quantum dots, rare-earth nanoparticles, and novel dyes—alongside continuous improvements in brightness, photostability, and biocompatibility. Imaging instrumentation has evolved from bulky benchtop InGaAs cameras to portable multispectral clinical systems, with endoscope-adapted NIR-II devices developed for specific clinical applications.

Importantly, NIR-I and NIR-II differ not only in wavelength but also in imaging performance and clinical maturity. NIR-I imaging is currently widely used in clinical practice due to its regulatory approval and well-established safety profile; however, it is limited by higher tissue scattering, stronger autofluorescence background, and relatively shallow imaging depth (29). In contrast, NIR-II imaging provides significantly improved spatial resolution, higher signal-to-background ratio, and deeper tissue penetration, making it more suitable for precise surgical navigation and deep tumor visualization (30). However, its clinical translation remains in an early investigational stage due to limited clinically approved probes and detector systems, with most current human studies relying on ICG tail-emission or early translational SWIR systems rather than dedicated NIR-II probes (31). The principle of NIR-II fluorescence imaging is straightforward: a molecular probe—either a dye or a nanoparticle—that emits light in the 1000–1700 nm wavelength range is injected into the patient. Once the probe has accumulated in the target tissue (such as a tumor) and been cleared from normal tissue, the region is illuminated to excite the probe and generate fluorescence. An imaging system with detectors sensitive to NIR-II light captures the emitted fluorescence. This produces a real-time image, showing where the probe has gathered—typically outlining the tumor and potentially revealing hidden tumor extensions or metastases. Because NIR-II light can penetrate deeper into tissues, this technique helps identify tumor regions that may not be visible on the surface, potentially warning the surgeon about unseen tumor growth (32).

In clinical practice, administration routes and timing depend strongly on the imaging agent and application scenario. For NIR-I clinical imaging, indocyanine green (ICG) is most commonly administered intravenously, typically minutes to 30 minutes prior to surgery for vascular perfusion or lymphatic mapping, and up to several hours for tumor-related applications depending on pharmacokinetics and target tissue characteristics (33). Topical or intracavitary administration, such as in gastrointestinal endoscopy or intraoperative cavity spraying, has also been reported, with imaging generally performed within minutes after application due to rapid local diffusion and tissue uptake (34). The safety profile of ICG is well established, with a very low incidence of adverse reactions and rapid hepatic clearance via biliary excretion (35). In contrast, most NIR-II fluorophores remain in preclinical or early translational stages and are generally administered intravenously in animal or early exploratory human studies, with imaging performed 4–24 hours (or longer) after administration to allow tumor accumulation via enhanced permeability and retention (EPR) effects or active targeting mechanisms (30). Safety evaluation remains a major barrier for clinical translation, particularly for nanoparticle-based probes, where long-term biodistribution, metabolic clearance, and immunogenicity remain incompletely characterized. Nevertheless, emerging small-molecule NIR-II dyes have demonstrated improved biocompatibility and faster clearance profiles in preclinical models, supporting their potential for future clinical translation (36).

NIR-II fluorescence imaging was first demonstrated *in vivo* in the late 2000s (**Figure 2**). A landmark early study by Hongjie Dai and colleagues in 2009 showed that single-walled carbon nanotubes (SWCNTs) could be used as fluorophores in the NIR-II range for deep-tissue imaging (37). In that work, the researchers were able to visualize blood vessels and organs several millimeters deep inside mice using SWCNTs emitting beyond 1000 nm. This was quickly followed by further proof-of-concept studies, such as the work of Welsher et al. (2011), which cemented the concept of a “second near-infrared window” for biomedical imaging (38). Over the past decade, the field of NIR-II imaging has witnessed rapid development, with researchers developing a variety of novel fluorophores—including inorganic nanomaterials and organic dyes—to fully exploit this optical window (32).

### 2.2 Evolution and development of NIR-II imaging technology

After initial work with SWCNTs, researchers began exploring other NIR-II probes to improve brightness, biocompatibility, and targeting. Early agents included quantum dots such as Ag_2_S emitting in the 1100–1300 nm range, and rare-earth doped nanoparticles that emit in NIR-II via downconversion (39, 40). These inorganic materials provided stable NIR-II signals and worked well for deep imaging in small animals. However, concerns about the toxicity of heavy-metal quantum dots and their long-term retention delayed their clinical use (41, 42).

On the organic side, scientists aimed to extend traditional NIR-I dyes into the NIR-II range. One approach was modifying polymethine cyanine dyes, such as ICG and IRDye800, to shift their emission past 1000 nm (43). Simply lengthening the polymethine chain can red-shift emission, but this often reduces dye stability and quantum yield. Thus, alternative approaches including altering the terminal heterocyclic moieties of the cyanine were pursued. This led to creation of dyes such as IR-1048 and IR-1061, and further innovations such as Flav7, a flavylium-based polymethine fluorophore with a quantum yield ~0.5 and emission extending past 1000 nm (44–46). Another dye, BTC1070, was developed by shortening the heptamethine chain and adding electron-donating groups; it achieved absorption and emission above 1000 nm with greatly improved photostability and brightness, delivering an intensity about seven-fold higher than the earlier IR-26 dye (47). These advancements indicated that purely organic small-molecule fluorophores could be engineered for the NIR-II window.

At the same time, new types of organic NIR-II emitters emerged, such as D-A-D conjugated dyes and aggregation-induced emission (AIE) fluorophores (48, 49). D-A-D dyes have electron-rich donor groups at both ends and an electron-deficient acceptor in the middle, producing fluorophores that often emit in NIR due to their charge-transfer excited states. By tuning donor and acceptor strength, emission can be pushed into NIR-II. For example, the dye CH1055 (named for its ~1055 nm emission peak) was one such D-A-D small molecule that found use in preclinical NIR-II imaging studies (50). AIE fluorophores, on the other hand, represent a newer paradigm in which fluorescence is weak when the molecules are isolated but becomes intense upon aggregation, in contrast to conventional dyes that suffer quenching when they aggregate (49). Researchers designed AIE nanoparticles with NIR-II emission, which benefit from high brightness due to the clustering of many fluorophores and avoidance of aggregation-caused quenching. These AIE dots and conjugated polymer nanoparticles have shown ultrabright NIR signals and are being explored for guided surgery as well (51).

The technology for NIR-II imaging also evolved. Initially, specialized InGaAs cameras that are sensitive to 900–1700 nm were required, but they were expensive and mostly found in research labs (52). In recent years, more portable and clinically friendly NIR-II imaging devices have been developed (53). For instance, an integrated multispectral imaging system was created that can simultaneously capture visible, NIR-I, and NIR-II fluorescence images during surgery (54). This allows surgeons to correlate what they see in white light with co-registered NIR fluorescence overlays. With the continued maturation of the technology, NIR-II cameras are becoming increasingly compact and easier to integrate into surgical workflows (**Figure 2**). In addition, some systems can be adapted into endoscopic platforms, enabling NIR-II imaging of internal organs (55).

### 2.3 Advantages of NIR-II imaging over NIR-I and other modalities

Deeper Tissue Penetration: Because of reduced light scattering and absorption at longer wavelengths, NIR-II photons can travel farther. Even a small improvement in penetration depth can be clinically significant; NIR-II can visualize structures or lesions that lie beyond the reach of NIR-I. In practical terms, NIR-I imaging might effectively penetrate on the order of 5–10 mm in tissue whereas NIR-II can often double that or more, enabling detection of deeper tumor margins or metastases (56, 57). For example, Shao et al. reported clear fluorescence imaging at 9 mm deep with NIR-II, whereas such depth would be challenging with NIR-I (56).

Higher Resolution at Depth: Light scattering in tissue is wavelength-dependent (Rayleigh scatter ~ 1/λ^4^). Thus, the longer wavelengths of NIR-II light scatter less, resulting in less image blur for objects at depth. The outcome is an improvement in spatial resolution for deep targets – fine structures can be distinguished more clearly in NIR-II images compared to NIR-I. Experiments have shown that NIR-II imaging yields sharper images of blood vessels and tumor margins even at several millimeters depth, where NIR-I images become fuzzy (58).

Better Signal-to-Background Ratio (SBR): Tissue autofluorescence is minimal in NIR-II, providing a very dark background (28). Additionally, many NIR-II probes have emission beyond the wavelengths where hemoglobin or water significantly fluoresce or autofluoresce (59). The combination of low background and strong targeted signals often yields SBRs that are significantly greater than those in NIR-I (60). For instance, imaging at 1300 nm has been shown to produce images with excellent contrast, enabling detection of tiny residual tumor deposits with high confidence (61). A recent comparison found that fluorescence imaging in NIR-II produced higher SBR and sensitivity than NIR-I in a clinical setting for liver tumor detection (54).

Reduced Tissue Absorption: Biological materials such as hemoglobin and melanin absorb much less light in the NIR-II range (above ~1000 nm) (59). This means the fluorescent light from a probe can exit the tissue without much loss, improving the detectable signal. It also means that even if there is some bleeding (which can quench visible light), the NIR-II signal might still come through (62), aiding surgeons in real scenarios where perfect hemostasis is not always maintained.

Potential for Multispectral Imaging: Because NIR-II is a broad window, in principle one could use multiple probes that emit at different sub-bands (say one at ~1050 nm and another at ~1300 nm) to image different targets simultaneously without spectral overlap. This “multiplexing” is more feasible in NIR-II because of the wide bandwidth available and the sharp optical filters for these ranges (63). This allows for the simultaneous imaging of different targets, such as tumors and critical structures, which can assist surgeons in making better intraoperative decisions.

Overall, NIR-II fluorescence imaging combines the benefits of fluorescence guidance—real-time feedback, high sensitivity, and easy integration with surgical tools—while overcoming some of the challenges of traditional NIR-I, especially in deeper tissues (64, 65). Early clinical studies show that NIR-II can help surgeons detect tumors with better sensitivity than NIR-I. The differences between the two fluorescence imaging modalities in surgical navigation are summarized in **Table 2**.

**Table 2:** Comparison of first and second near-infrared window fluorescence imaging for surgical guidance.

Feature	NIR-I (650–900 nm)	NIR-II (1000–1700 nm)
Tissue penetration depth	Moderate (few mm up to ~10 mm in ideal conditions) (66)	Higher (potentially up to 20–30 mm under ideal conditions); photons travel farther due to less scatter (66, 67)
Spatial resolution at depth	Limited by stronger scattering; deeper structures appear blurred (67).	Improved resolution at depth (reduced scattering yields sharper images). Fine structures (vessels, margins) remain clearer (67).
Autofluorescence background	Present (tissue autofluorescence from proteins, lipofuscin, etc., in visible/NIR-I range) (68).	Negligible autofluorescence; very low background in biological tissues, enhancing contrast (68).
SBR	Good (SBR can be high for surface lesions but drops with depth or in fatty tissue) (26).	Typically higher SBR than NIR-I for the same target. Yields excellent contrast even for deeper or small lesions (26).
Examples of clinical fluorophores	ICG; IRDye800CW; 5-ALA (visible, 635 nm, for brain tumors). These emit in or near NIR-I (64, 69).	Emerging: ICG (tail emission in NIR-II); novel cyanine derivatives (e.g., IR-1061); CH1055 dye; Quantum dots (Ag_2_S, etc.); rare-earth nanoparticles. (Most not yet clinically approved, but used in research and trials.) (39, 50, 70–73)
Notable uses	A seminal first-in-human study demonstrated that real-time folate receptor-α-targeted fluorescence imaging using folate-FITC during ovarian cancer cytoreductive surgery enables visualization of tumor deposits not apparent by conventional inspection alone (74); Sentinel lymph node mapping and angiography with ICG; Margin detection in clinical trials utilizing IRDye800-labeled tumor-specific antibodies in surgical studies (75, 76)	High-resolution tumor imaging in research and early trials (e.g., liver tumor resection with ICG in NIR-II mode; NIR-II endoscopy for GI lesions in trial). Enables detection of residual tumor after standard resection in cases where NIR-I might miss it (77, 78)

## 3 NIR-II fluorescence molecular probes: development and clinical applications

The successful implementation of NIR-II fluorescence imaging in surgery critically depends on the availability of effective molecular probes. Specifically, these probes should be capable of selectively accumulating in tumor tissue or labeling surgical targets, while emitting fluorescence in the NIR-II window. Over the past decade, a wide range of NIR-II probes have been extensively developed, spanning from organic small-molecule dyes to inorganic nanoparticles. These probes can be broadly classified into two major categories—non-targeted and targeted—and representative applications have been reported across diverse organ systems and disease settings.

### 3.1 Categories of NIR-II fluorescent probes for image-guided surgery

#### 3.1.1 Non-targeted or passive probes

Non-targeted NIR-II probes accumulate in tumors through general mechanisms via the enhanced permeability and retention (EPR) effect or vascular mapping, rather than targeting specific molecules (79). These probes typically highlight tumors due to leaky blood vessels or poor lymphatic drainage, or by showing blood supply and tissue structure (80).

A common non-targeted probe is ICG, an FDA-approved dye used in the NIR-I range (~820 nm) for imaging and liver function tests (81). Interestingly, ICG also emits light in the NIR-II range (900-1000+ nm) when it binds to plasma proteins (28). This makes ICG useful for NIR-II imaging, and several studies have repurposed it for this purpose. ICG clears through the liver within about 6–12 hours and does not inherently target tumors, but it can still outline tumors via perfusion differences or temporary retention (82). In a study, it was demonstrated that injection of ICG 24 hours later can clearly delineate tumor margins with a maximum~8.39:1 SBR under NIR-II imaging conditions (83). Another erythrocyte-based probe strategy involved loading ICG onto red blood cell membranes and converting it into nanoparticles, thereby prolonging its blood circulation time. In a murine liver tumor model, these erythrocyte–ICG nanoparticles exhibited prominent NIR-II tumor fluorescence within an approximately 10-hour “surgical window” after injection, with the SBR stably maintained at 11.5 (84). These data suggest that even without active targeting, ICG in NIR-II mode can provide useful contrast for tumors, especially hepatic lesions, given the liver’s role in ICG metabolism.

Other non-targeted probes include various polymer-based NIR-II nanoparticles and quantum dots that passively accumulate in tumors. For instance, Ag_2_S quantum dots emitting at ~1100 nm have been used to visualize tumors and lymph nodes in mice due to passive accumulation (85, 86). They are sufficiently small, with a diameter of approximately 10–15 nm, enabling effective exploitation of the EPR effect (87). Similarly, some AIE nanoparticles loaded with NIR-II AIEgen molecules can flood tumor tissue with bright emission simply by prolonged circulation (88). These probes often rely on the tumor’s abnormal vasculature rather than specific molecular recognition. The primary advantage of non-targeted probes lies in their broad applicability and independence from specific receptors or biomarkers. These agents typically accumulate within the tumor stroma or leaky vasculature, thereby delineating the bulk tumor region. However, their major limitation is the relatively low specificity, often resulting in off-target uptake by the liver, spleen, or inflammatory sites. Furthermore, they generally exhibit higher background signals in normal tissues compared to targeted probes.

Non-targeted NIR-II imaging can also be applied intraoperatively to guide resection by mapping vascular architecture or perfusion patterns. In certain surgical procedures, it is essential not only to identify tumors but also to preserve or localize critical normal anatomical structures. For instance, in reconstructive microsurgery, surgeons have utilized NIR-II ICG imaging to identify perforator vessels and assess flap perfusion (89). Non-targeted NIR-II probes, such as ICG, serve as versatile contrast agents playing a pivotal role in the clinical setting. Given that agents such as ICG are already clinically approved, their application requires only the integration of NIR-II imaging devices, making them the preferred choice for initial clinical trials. Preliminary evidence suggests that NIR-II imaging can significantly enhance surgical precision. However, the future of precision surgical imaging likely lies in the development of more targeted probes.

#### 3.1.2 Targeted and activatable probes

Targeted NIR-II probes are designed to bind specifically to tumor cells or the tumor environment, typically by attaching a targeting molecule to a fluorescent dye (90). This targeting molecule could be a monoclonal antibody, a fragment (Fab or nanobody), a peptide, a small molecule, or an aptamer that recognizes a tumor-specific marker. The goal of these probes is to improve tumor detection, especially for cancers that are diffuse or infiltrative, where non-targeted probes might not be enough.

One example is a probe targeting the EGFR protein in head and neck or brain tumors. IRDye800CW, a NIR-I dye, was conjugated with cetuximab (an anti-EGFR antibody) and used to highlight EGFR-positive tumors such as oral cancers (91). While IRDye800CW is primarily NIR-I, similar concepts are being translated to NIR-II by either using antibodies with new NIR-II dyes or detecting the tail of IRDye800 in NIR-II. In fact, one recent colorectal cancer study used a CEACAM5-targeted antibody (named 2D5) conjugated with IRDye800CW and performed imaging in the NIR-II window (92). The probe specifically bound colorectal tumor cells expressing the CEA antigen and allowed visualization of tumors during surgery with high contrast. This indicates that even probes developed for 800 nm can be utilized in extended spectrum imaging, although truly optimized NIR-II fluorophores attached to antibodies are in development.

Peptide-based targeting is also common. Urokinase plasminogen activator receptor (uPAR) is overexpressed in many aggressive cancers such as glioblastoma (93). Kurbegovic et al. developed a peptide (AE105) that binds uPAR and conjugated it to a NIR-II dye CH1055, yielding CH1055-4Glu-AE105 (94). In an orthotopic mouse glioblastoma model, this targeted probe enabled clear delineation of tumor boundaries under NIR-II imaging. The researchers further quantified the margin-to-background ratio (MBR)—a metric analogous to SBR but specifically designed to compare fluorescence intensity at the resection margin with that of normal brain tissue—to assess the completeness of resection. An MBR value of 1.0 indicates the absence of residual tumor, whereas a higher MBR, such as 1.5, suggests incomplete resection (94). This quantitative targeted imaging strategy holds substantial translational value in the operating room, allowing surgeons to directly visualize microscopic residual tumor at the margins and take additional measures when necessary.

Enzyme-activated probes are another targeted approach. These probes remain “off” until they encounter a tumor-specific enzyme. In a renal cell carcinoma model, An et al. designed a peptide probe cleavable by tumor-associated matrix metalloproteinases MMP2/9. The probe contains a self-quenched near-infrared fluorophore that, after enzymatic cleavage in the tumor, is released and assembles into fluorescent nanoparticles on the tumor cell surface. The design emits fluorescence only where enzymes are active, enhancing specificity and improving contrast by minimizing off-target signals. Results showed that this MMP-activated NIR probe clearly delineated renal tumor margins and detected satellite lesions smaller than 1 mm that would otherwise be missed (95). While their probe was NIR-I, similar designs are being pursued in NIR-II. These activatable NIR-II probes, designed to respond to factors such as tumor pH, glutathione levels, or enzyme activity, may further enhance tumor-to-normal tissue contrast. This is because the background signal in healthy tissue remains nearly zero until the probe is specifically “switched on” by the tumor itself (96).

Small-molecule targeting ligands have also been explored. One example is a HDAC6-targeted small molecule probe for hepatocellular carcinoma (HCC). HDAC6 is a cellular enzyme overexpressed in many HCC tumors (97). Bo Wang et al. attached a HDAC6 inhibitor moiety to IRDye800CW to create SeCF_3_-IRDye800, which emits in the NIR-I but they utilized its NIR-II range emission for imaging (98). In preclinical models, the targeted probe showed much higher uptake in HCC tumors compared to non-targeted ICG, and it illuminated residual tumor deposits during simulated surgery. They also incubated the probe with human HCC surgical specimens ex vivo and demonstrated it could label microscopic tumor foci on resected tissue. This kind of small-molecule targeting is attractive because the probes are usually cleared faster, which can potentially yield a better tumor-to-normal ratio in a shorter time, and they may penetrate tissue more uniformly than larger antibodies.

Interestingly, some NIR-II probes achieve targeting not through biological ligands but by exploiting tumor physiology. For instance, certain meso-chlorine substituted heptamethine cyanine dyes tend to bind serum albumin and accumulate in tumors without any specific “lock-and-key” receptor binding (99). These dyes—often referred to as Tumor-Targeting Cyanines, such as IR-780 or ZW800–1 derivatives—localize in tumors through a mix of the EPR effect and the strong uptake of albumin by cancer cells (100). Essentially, they provide passive targeting with a chemical tweak that improves tumor selectivity. Such dyes in NIR-II form, with emission beyond 1000 nm, are also being explored (101).

Targeted NIR-II probes are an exciting and rapidly evolving area of research. Their main strength lies in their specificity — by focusing on cancer’s unique molecular markers, these probes allow tumors to be visualized more precisely with minimal background interference. This accuracy is especially important for defining tumor margins, since nearby healthy tissue can sometimes take up nonspecific probes. With targeted imaging, ideally only the remaining tumor tissue lights up, giving the surgeon a clear signal of where additional removal may be needed. Referring to the recently published guidelines for fluorescent molecular imaging studies jointly issued by WMIS and ESMI (102), we have listed over ten typical NIR-II molecular probes in **Table 3**, providing a detailed summary of their standardized characteristics, routes of administration, imaging parameters, and other relevant information.

**Table 3:** Standardized characteristics, translational status, administration, and imaging parameters of probes used for NIR-II/SWIR-assisted image-guided surgery.

A. Probe identity, spectral class, targeting, and translational status						
Probe (type)		Emission maximum and spectral classification	Targeting or mechanism		Evidence level/regulatory status	Refs
ICG (small-molecule cyanine dye)		~820 nm peak (NIR-I); long-wavelength tail detected in NIR-II/SWIR (>1000 nm). NIR-I-peak/NIR-II-tail probe, not a true NIR-II-peak dye	Non-targeted; plasma-protein binding, vascular perfusion and hepatic handling; tumour contrast is not receptor-mediated		ICG is clinically approved for conventional indications. Human NIR-II/SWIR imaging in Refs. 49/82 was an off-label use of its tail emission	(54, 89)
6PEG-Ag2S quantum dots (inorganic nanoparticle)		~1200 nm (true NIR-II peak)	Non-targeted; passive tumour accumulation consistent with prolonged circulation/EPR		Preclinical animal study only; no clinical approval. Translation is limited by nanoparticle composition, long-term safety and manufacturing issues	(85)
CH1055-PEG (D-A-D organic small-molecule fluorophore)		1055 nm (true NIR-II peak)	Non-targeted PEGylated dye; passive tumour uptake; can be further functionalized.		Preclinical only; not clinically approved.	(50)
CH1055-4Glu-AE105 (targeted peptide-dye conjugate)		1055 nm (true NIR-II peak)	Active targeting of urokinase-type plasminogen activator receptor (uPAR) by AE105 peptide.		Preclinical orthotopic brain-tumour study; not clinically approved.	(94)
Cetuximab-IRDye800CW (antibody-dye conjugate)		~789–800 nm (NIR-I peak)	EGFR-targeted monoclonal antibody conjugate.		Clinical phase-I investigation in head-and-neck cancer; the conjugate was investigational, not an approved imaging agent.	(69)
SeCF3-IRD800 (HDAC6-targeted small-molecule conjugate)		IRDye800-based NIR-I emission peak; signal acquired in NIR-II using the long-wavelength tail (>=1000 nm). NIR-I-peak/NIR-II-tail probe.	Active targeting of histone deacetylase 6 (HDAC6) in hepatocellular carcinoma.		Preclinical mouse study plus ex-vivo human HCC specimens; no in-human administration and no clinical approval.	(98)
IR1061-loaded AuNR@PEG/PolyRu vesicles (AuNR Ves)		IR1061-centred NIR-II emission (~1060 nm; true NIR-II fluorophore within a nanovesicle formulation).	Non-targeted/passive tumour accumulation; 808-nm light triggered vesicle disruption and cargo release.		Preclinical MCF-7 xenograft theranostic study; not clinically approved.	(103)
Lf@IR-1048 (lactoferrin-docked cyanine formulation)		1048 nm (true NIR-II peak).	Lactoferrin-mediated active tumour targeting, associated with transferrin-receptor-1 recognition.		Preclinical CT26 tumour study; not clinically approved.	(104)
A1 nanoparticles (AIE nanoparticle)		1050 nm (true NIR-II peak).	Non-targeted/passive tumour accumulation.		Preclinical orthotopic 4T1 breast-tumour model; not clinically approved.	(105)
DNA/FSHbeta-functionalized NaGdF4:Nd downconversion nanoparticles (DCNPs)		1060 nm under 808-nm excitation (true NIR-II emission).	FSH-receptor targeting plus sequential complementary-DNA-mediated *in-vivo* assembly to increase tumour retention.		Preclinical metastatic ovarian-cancer study; not clinically approved.	(106)
ICG-C9 and ICG-C11 cyanine probes/bioconjugates		ICG-C9: 922 nm (transition/SWIR-tail region); ICG-C11: 1010 nm (true NIR-II/SWIR peak) in water.	Structures were conjugated to biomolecules for multiplexed molecular targeting; targeting depended on the selected bioconjugate.		Preclinical *in-vivo* mouse molecular-imaging study; neither probe is clinically approved.	(107)

B. REFLECT-recommended administration and image-acquisition parameters						
Probe/study	Route and formulation	Administered dose	Administration-to-imaging/surgery interval	Imaging system and acquisition parameters	Key outcome/reporting note	Refs
ICG - liver-tumour surgery	Intravenous ICG.	0.5 mg/kg	1–7 days before surgery	Integrated multispectral visible/NIR-I/NIR-II system; concurrent comparison of visible, NIR-I and NIR-II channels	Human liver-tumour surgery; NIR-II improved sensitivity/contrast relative to NIR-I in the study. ICG use in NIR-II was off-label	(54)
ICG - microsurgery	2 ml ICG intravenously; For the perforator-flap subgroup, concentration was 2.5 mg/ml	2 ml per imaging episode. For the perforator-flap subgroup: 5 mg total	Imaging commenced immediately after injection; avulsion injury was assessed at 1, 3, 5 and 10 min; real-time angiography was used in other procedures	Portable system: NIR-II 900–1700 nm InGaAs camera (640x512), cooled to -80 C, 50-mm lens; NIR-I silicon camera with 800-nm long-pass and 900-nm short-pass filters; 785-nm excitation at 10–15 mW/cm2; working distance 20–40 cm for flap imaging	39 patients. Reported uses included anastomotic patency, digit perfusion/venous drainage, avulsion viability and perforator-flap planning	(89)
6PEG-Ag2S QDs	Tail-vein intravenous injection in mice	200 microliter of 1.34 mg/ml	Dynamic imaging immediately after injection and serial imaging from minutes to 24 h.	808-nm excitation with NIR-II detection using an InGaAs-based setup; full settings should be cited from the original methods	Preclinical only; prolonged circulation, tumour uptake and hepatobiliary clearance were demonstrated	(85)
CH1055-PEG	Intravenous administration for brain-tumour imaging; intradermal administration was used in a separate lymphatic-mapping experiment.	approximately 10 microliter of 300 micromolar (~3 nmol) intradermally.	Brain-tumour imaging was reported at 6 h, with whole-body clearance assessment up to 24 h; SLN imaging commenced immediately.	NIR-II InGaAs imaging; the SLN experiment used 1200-nm long-pass detection and 200-ms exposure	The paper supports rapid renal excretion and deep preclinical imaging, but not clinical use.	(50)
CH1055-4Glu-AE105	Tail-vein intravenous injection in mice	40 nmol per mouse	Serial imaging; maximum TBR approximately 2.7 at 96 h, which was used for image-guided surgery	750-nm excitation with NIR-II detection of CH1055 emission; detailed detector/filter settings should be retained from the source methods.	Targeted preclinical probe; delayed optimal imaging reflects the pharmacokinetics of the targeting conjugate.	(94)
Cetuximab-IRDye800CW	Intravenous infusion in patients.	Dose-escalation cohorts: 2.5, 25 and 62.5 mg/m^2^.	Surgery/imaging 3–4 days after infusion.	Clinical NIR-I fluorescence imaging with multiple optical instruments, but no NIR-II acquisition	Phase-I head-and-neck cancer study. Report as an investigational targeted NIR-I tracer, not an approved NIR-II probe.	(69)
SeCF3-IRD800	Tail-vein intravenous injection; formulation concentration reported as 0.3 mg/ml	10 mg/kg (mouse I)	Optimal tumour imaging approximately 8 h after injection.	808-nm excitation; fluorescence collected with a 1000-nm long-pass filter; exposure approximately 300 ms.	Preclinical HCC surgery; renal clearance. Human validation was limited to freshly resected ex-vivo HCC tissue.	(98)
IR1061-loaded AuNR Ves	Tail-vein intravenous injection	200 microliter of 4 mg/mL vesicles (0.8 mg formulation/mouse)	Serial imaging 6–36 h; strong tumour accumulation and light activation were evaluated at approximately 24 h.	NIR-II fluorescence under 808-nm excitation; approximately 1150-nm band-pass detection and 900-ms exposure. Activation: 808 nm, 50 mW/cm2 for 5 min.	Mass dose refers to the composite vesicle formulation and should not be presented as an IR1061 molar dose.	(103)
Lf@IR-1048	Intravenous injection	IR-1048-equivalent dose: 2 mg/kg	Imaged at 6, 12, 24 and 48 h; tumour signal peaked at approximately 12 h	MARS NIR-II imaging system; 980-nm excitation was used for *in-vivo* imaging	One methods paragraph states intratumoral injection, whereas the results description states intravenous administration; the table follows the whole-body imaging description and flags the inconsistency	(104)
A1 nanoparticles	Tail-vein intravenous injection	NR	Serial imaging; maximum TBR 3.8 +/- 0.35 at 24 h, selected for surgery.	808-nm excitation with a custom NIR-II system; exact detection band/exposure: NR	Residual tumors as small as approximately 2 mm and metastatic lymph nodes were identified in the preclinical model.	(105)
DNA/FSHbeta-functionalized DCNPs	Sequential tail-vein injections of two nanoprobe	7.5 mg/kg for the first component and 7.5 mg/kg for the second component 8 h later.	Second injection at 8 h; stable surgical window approximately 20–28 h after the first injection	808-nm excitation at 40 mW/cm2; 1000-nm long-pass detection; working distance 30 cm	*In-vivo* assembly enhanced tumour retention and enabled fluorescence-guided resection of metastatic ovarian lesions	(106)
ICG-C9/ICG-C11 bioconjugates	*In-vivo* administration route/formulation depended on the selected bioconjugate.	NR	0–24 h (optimal 4–12 h)	NR	SWIR-emitting cyanine probes enabling multiplexed NIR-II imaging	(107)

a “NIR-II-tail detection” indicates that the fluorophore has an emission maximum in NIR-I but its weak long-wavelength tail is collected above 1000 nm. This must be distinguished from a fluorophore whose emission maximum lies in NIR-II.

b. ICG is clinically approved, but its use for NIR-II/SWIR tail-emission imaging is off-label. Cetuximab-IRDye800CW was investigational in Ref. 63. None of the true NIR-II-peak probes summarized here was approved for clinical use in the cited studies.

c. Parameters not explicitly available in the cited article were recorded as NR.

#### 3.1.3 Key considerations for probe development

Probe Delivery and Timing: Practical use of NIR-II probes also depends on how and when they are administered. Some agents, such as antibody-based probes, may need to be given 1–3 days before surgery to achieve optimal tumor accumulation (69). Others such as ICG can be administered several hours prior to surgery (108). The ideal probe would enter tumors quickly, clear from normal tissues rapidly, and offer a strong signal at the time of surgery. However, many NIR-II probes, particularly nanoparticles, stay in circulation for long periods, which can raise safety questions (109). Although highly effective for imaging, prolonged probe retention raises important concerns regarding safety and clinical applicability. A probe that remains in the body for weeks may induce adverse effects and could interfere with postoperative imaging modalities such as MRI or PET (110). Therefore, biocompatibility and clearance of probes are under scrutiny. To address this, researchers are developing smaller, kidney-clearable probes — such as organic dyes under 2 kDa or nanoparticles smaller than 5 nm — that the body can safely eliminate (111).

Target Selection: Choosing the right molecular target remains a cornerstone of developing effective NIR-II probes. A good target should be highly expressed in tumor tissue but largely absent in healthy cells, and ideally be accessible on the cell surface. Common choices include EGFR, HER2, CEA, the folate receptor, and PSMA (112). However, since tumors can vary widely between patients, a single probe is unlikely to work for everyone. Personalized approaches, in which probes are selected based on a patient’s tumor profile, may represent the next frontier of surgical precision (98). On the other hand, more general probes, including albumin-binding cyanine dyes that accumulate in many tumors, could serve as “universal” options, though with slightly less specificity (113).

### 3.2 Applications of NIR-II imaging probes in different surgical domains

#### 3.2.1 Brain tumor (neurosurgery)

Complete resection of malignant brain tumors, exemplified by high-grade gliomas, is associated with improved survival, but achieving clear margins is difficult due to the infiltrative nature of glioma cells and the need to preserve eloquent brain tissue (114). Visible-light fluorescence using 5-ALA causes protoporphyrin IX to accumulate in tumors and is an approved technique for glioblastoma surgery, but its shallow penetration and subjective interpretation leave room for improvement (115). NIR fluorescence could penetrate the brain tissue a bit further; indeed, NIR-I probes including IRDye800CW-labeled antibodies have been tested in brain tumor patients, for example, to highlight glioma or meningioma margins (116). NIR-II imaging now offers an even more attractive option. In preclinical studies, NIR-II probes have shown the ability to delineate brain tumor boundaries with unprecedented clarity. For example, the uPAR-targeted CH1055-4Glu-AE105 discussed earlier allowed researchers to distinguish tumor from normal brain in a mouse glioblastoma model with high precision (**Figure 3B**) (94). These findings suggest that NIR-II probes could significantly assist neurosurgeons in achieving maximal safe resection, especially for infiltrative tumors. While no NIR-II agents have yet reached routine clinical use in neurosurgery, clinical trials are on the horizon. Researchers are actively investigating molecular targets such as EGFR, which is markedly upregulated in multiple types of gliomas. For instance, a preclinical study evaluated the pharmacological characteristics and safety profile of the probe ABY-029 in animal models (117). This probe is composed of a fluorescently labeled anti-EGFR Affibody molecule conjugated to IRDye800CW. The study provided essential data to support subsequent phase 0 clinical trials in humans and established a preclinical rationale for fluorescence-guided glioma resection. It can be anticipated that next-generation NIR-II imaging probes will emerge accordingly. The blood-brain barrier is a consideration for systemic probe delivery, but many gliomas disrupt this barrier, allowing probe accumulation (118). In summary, NIR-II fluorescence holds promise to enhance intraoperative visualization of brain tumors, potentially increasing resection extent while minimizing neurological deficits by providing real-time feedback on where tumor ends and normal brain begins (**Figure 3**).

**Figure 3:**
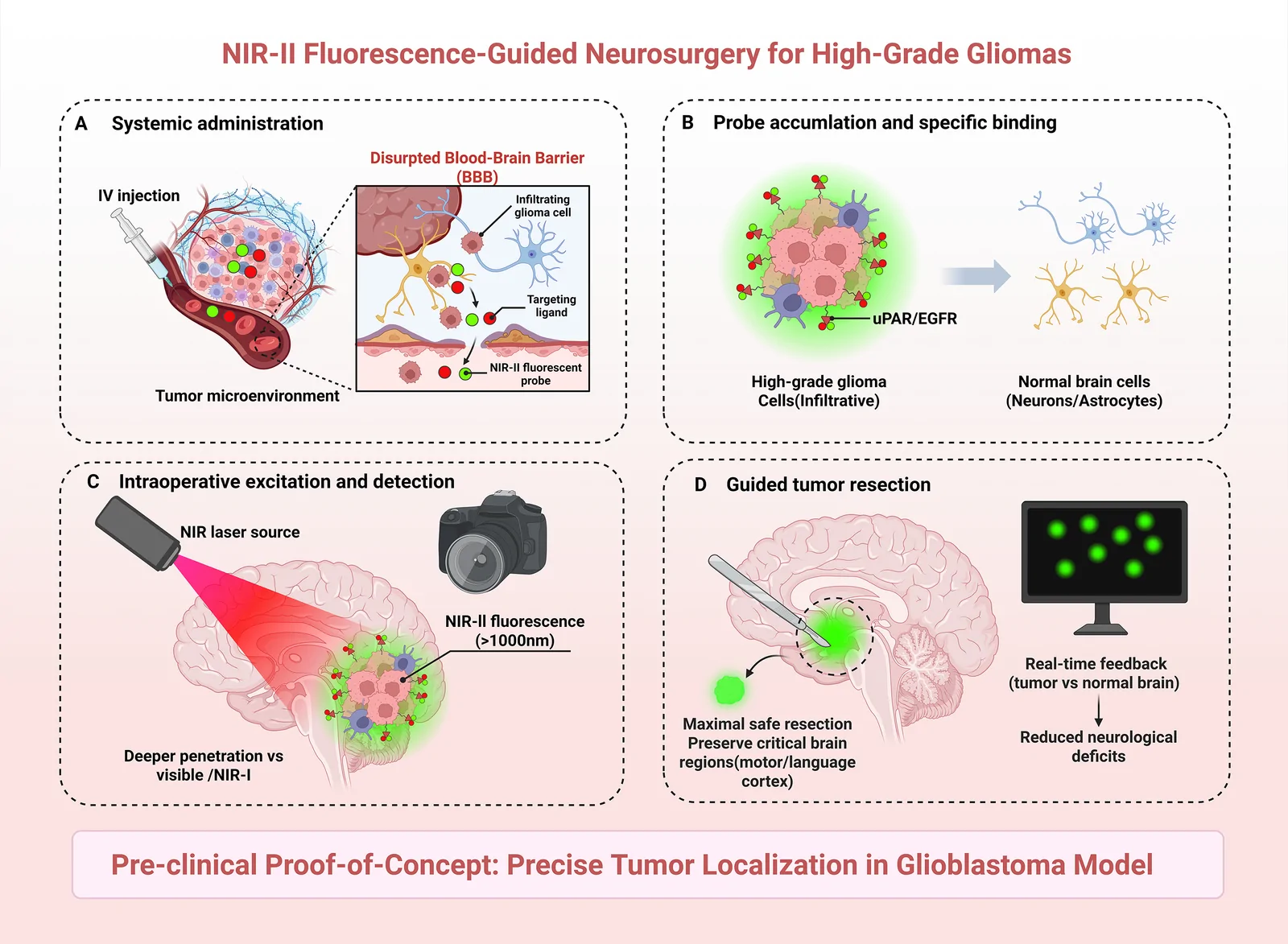
Schematic illustration of preclinical validation for precise tumor localization in a glioblastoma model. Following intravenous administration, the NIR-II probe penetrates the blood-brain barrier (BBB) and selectively binds to highly expressed targets on tumor cells within the tumor microenvironment, while sparing normal cells **(A, B)**. Intraoperative laser excitation elicits NIR-II fluorescence emission, delineating tumor margins with a bright outline against a dark background and demonstrating deep tissue penetration **(C)**. The surgeon subsequently resects the tumor along the fluorescent margins to ensure maximal safe resection and real-time differentiation between tumor and normal brain tissue **(D)**.

#### 3.2.2 Head and neck surgery

Head and neck tumors, such as those in the oral cavity, pharynx, or thyroid, involve regions rich in critical nerves and blood vessels. As a result, complete tumor resection is required, but it is equally important to preserve the function and appearance of the surrounding tissues. If the surgical margins are unclear, the risk of tumor recurrence significantly increases. Traditional methods such as palpation and frozen section analysis have limitations, often necessitating additional surgeries to excise residual tumor (119). Fluorescence imaging has begun to make inroads here: one notable success has been sentinel lymph node mapping using ICG in oral and thyroid cancers to identify nodes at risk (120, 121). This is NIR-I based and improves staging accuracy. In the future, NIR-II fluorescent probes specific to head and neck tumors could further refine surgical precision. Preclinical studies have tested agents such as an EGFR-targeted antibody-dye conjugate in orthotopic tongue cancer models, demonstrating that fluorescence can highlight cancerous mucosal margins that are otherwise occult (122). A systematic clinical comparison between NIR-I and shortwave-infrared/NIR-II spectral imaging in head and neck and other tumor specimens has demonstrated that SWIR/NIR-II imaging can significantly improve tumor-to-background contrast and boundary delineation compared with conventional NIR-I systems, particularly in clinical tumor tissue containing cetuximab-IRDye800CW probes (123). This provides direct clinical evidence supporting the superior imaging performance of longer-wavelength fluorescence imaging in surgical oncology. If these probes are adapted for use in the NIR-II range, the improved contrast and imaging depth could make it possible to detect deep margins or perineural spread during surgery. Head and neck cancers often show strong inflammation, especially after radiation therapy. Interestingly, some targeted fluorophores have been shown in animal studies to differentiate cancerous tissue from inflammation (124). Because NIR-II imaging offers higher contrast with less nonspecific uptake and autofluorescence, it could help reduce false-positive signals caused by inflammation. Although specific NIR-II probes for head and neck tumors are still in preclinical development, it is conceivable to adapt agents including panitumumab-IRDye800—an anti-EGFR probe already evaluated in oral cancer trials—by pairing it with a second-window dye to enhance its performance (125). Another promising target is the folate receptor, which is often overexpressed in certain oral cancers and has already been used clinically with a NIR-I folate-FITC probe (126). Developing second-window folate dyes could be the logical next step. Overall, NIR-II fluorescence-guided surgery has the potential to greatly benefit head and neck oncology by allowing surgeons to visualize tumor boundaries more clearly in real time—crucial when operating near nerves, salivary glands, or vocal structures.

#### 3.2.3 Breast cancer surgery

Breast-conserving surgery for breast cancer requires accurate tumor localization and margin assessment to minimize re-excisions. Currently, about 20% of patients require a second surgery due to positive margins (127). Imaging the tumor intraoperatively is challenging – specimen X-rays and touch-prep cytology are used, but real-time visualization of residual tumor in the cavity could dramatically reduce positive margins. Fluorescence technology is currently being widely researched. For example, a clinical trial evaluated BLZ-100, a conjugate of chlorotoxin-based peptide and visible fluorophore, for its feasibility in fluorescence-guided intraoperative margin assessment (128). The researchers successfully detected small tumors with a mass as low as 0.5 mg by covalently conjugating a monoclonal antibody against HER2-positive breast cancer with IRDye800 (129). Continuous surgical resection results indicated that real-time fluorescence imaging could distinguish ligand-specific binding in areas of the lesion that were not yet visible clinically. These studies fall within the NIR-I imaging range, and the transition to NIR-II imaging is expected to further enhance the detection capability of small tumor lesions. An innovative probe, TPA-TQ3 aggregation-induced emission nanoparticles, was applied in a mouse breast tumor model and, despite emitting at approximately 800 nm in the NIR-I range, enabled unambiguous *in vivo* detection of sub-millimeter tumor nodules by fluorescence imaging during surgery. The fluorescence signal showed precise spatial concordance with bioluminescent tumor cells, verifying that the detected foci were bona fide tumor deposits. Although confined to NIR-I emission, the probe’s strong diagnostic performance indicates that shifting into the NIR-II window, either through similar AIE structural optimization or by harnessing tail emission, could provide greater imaging depth and clarity (130). Another promising agent is VGT-309, an integrin-targeted NIR fluorophore (in trials as NIR-I) – early data in breast surgical models show it lights up residual tumor in the resection cavity, guiding further excision (131). Future NIR-II versions of such targeted dyes might detect residual cancer cells at the margins that would otherwise be invisible. Beyond the primary tumor, NIR-II imaging could assist in identifying involved lymph nodes (132). ICG is already used for sentinel lymph node mapping in breast cancer, typically detected in the NIR-I window (133). If surgeons use an InGaAs camera and appropriate filters, the same ICG injection can be viewed in NIR-II (89), which might reveal nodes hidden behind fatty tissue or at greater depths with higher contrast.

Breast cancer guidelines recommend biopsy of all suspicious regional lymph nodes for preoperative assessment; however, limitations of sampling techniques make it challenging to accurately detect and evaluate microscopic lesions smaller than 0.5 cm (134). Alexander L Antaris et al. achieved the imaging of lymphatic vasculature, lymph nodes, and lymphatic drainage tumors in U87MG tumor-bearing mice, and for the first time demonstrated that mice injected with the CH1055-PEG molecular fluorophore in the NIR-II window exhibited superior image clarity, spatial resolution, and penetration depth compared with the FDA-approved NIR-I molecular fluorophore (50). Notably, some researchers have even extended the imaging range to the NIR-IIb window 1500–1700nm; for example, Yang et al. synthesized an NIR-IIb rare earth nanoprobe, RENPs@HA, for the real-time detection and resection of Sentinel lymph node metastasis in breast cancer, which accurately determined lymph node status with high sensitivity and specificity and enabled the detection of macro- or micrometastases with great sensitivity (135). This methodology holds promise for the precise visualization of lymph node metastasis, thereby enhancing the advantages of early tumor staging and treatment decision-making. Although these are early-stage observations, they hint that NIR-II could provide an edge in breast surgery, where tissue optics can limit visible/NIR-I light penetration. We anticipate clinical trials in the next few years applying NIR-II probes for margin guidance in breast lumpectomy – an advance that could lower re-operation rates significantly.

#### 3.2.4 Hepatobiliary and pancreatic surgery

Liver and pancreatic cancers are among the most challenging tumors to treat surgically. During liver resections, surgeons must identify tumors, whether they are small and multiple as in colorectal metastases or diffuse within cirrhotic tissue as in hepatocellular carcinoma, while securing clear margins and preserving enough healthy liver for function (136, 137). In pancreatic cancer, particularly pancreatic ductal adenocarcinoma, positive margins are common because of the dense fibrotic stroma and the tumor’s tendency for early perineural invasion (138). Although intraoperative ultrasound remains the standard imaging tool in these operations, fluorescence imaging is gaining attention as a valuable complementary approach (**Figure 4**).

**Figure 4:**
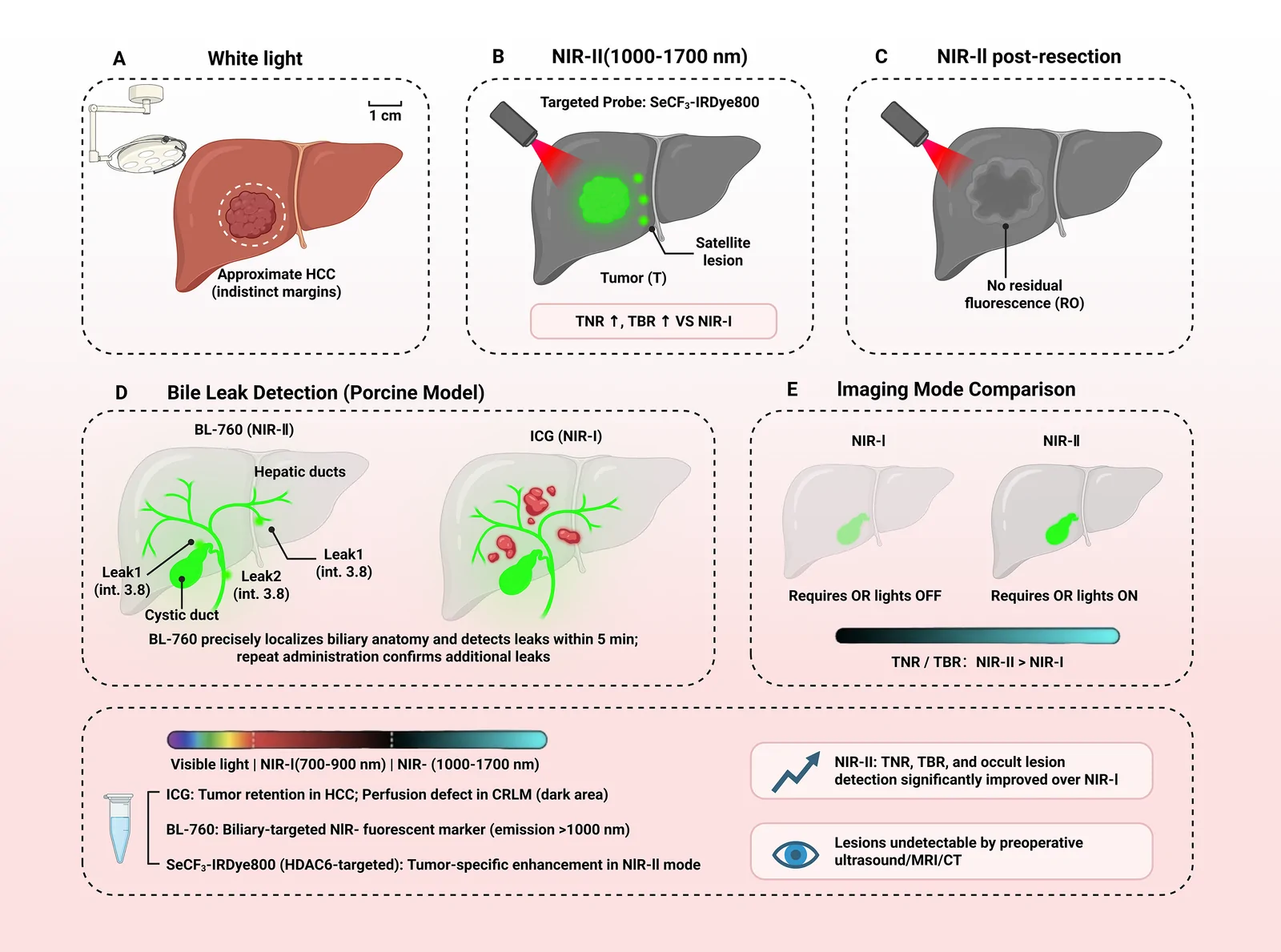
NIR-II fluorescence-guided detection of residual disease in surgery. White-light imaging view: The surgical perspective under conventional illumination shows indistinct tumor margins with poor differentiation between the tumor and surrounding liver tissue near the dashed line **(A)**. NIR-II fluorescence imaging of the same field after administration of the HDAC6-targeted probe (SeCF_3_-IRDye800): The tumor margins are clearly delineated. A small satellite lesion, invisible under white light, is revealed solely by the fluorescence signal **(B)**. Post-resection NIR-II imaging: No residual fluorescence signal is detected in the liver remnant, confirming a complete (R0) resection. Without fluorescence guidance, such microscopic residual lesions might have been overlooked **(C)**. Comparison in a porcine bile leak model: BL-760 under NIR-II imaging clearly visualizes three leakage sites. In contrast, ICG under NIR-I imaging fails to distinguish these sites due to high background signal from hepatic parenchyma and obstruction by blood oozing **(D)**. Direct comparison of the same lesion under NIR-I versus NIR-II modes: NIR-I: Higher background brightness, lower contrast, and the necessity to turn off ambient surgical lights. NIR-II: Superior contrast, increased penetration depth, and reduced light scattering **(E)**. Schematic illustration based on Ref (54).

In liver cancer, ICG has achieved notable success as a near-infrared imaging agent. When administered intravenously a few days before surgery, ICG is selectively retained in liver tumors—hepatocellular carcinomas often fluoresce because of impaired biliary excretion, while colorectal metastases typically appear as dark “holes” against the surrounding fluorescent liver tissue due to perfusion defects (139). Surgeons in Asia have widely adopted ICG fluorescence to find small liver tumors and to demarcate liver segment boundaries during resection (140). Preclinical studies in porcine models have validated the application of biliary-targeted fluorophore BL-760 (>1000 nm emission in the NIR-II window) in hepatic surgery. In a 2019 study, researchers performed laparoscopic cholecystectomies and left lobectomies in pigs, where BL-760 clearly delineated the gallbladder, cystic ducts, and intrahepatic bile ducts, enabling detection of bile leakage during hepatectomy (25).

A follow-up investigation focused on segmental hepatectomies in 25–30 kg pigs, comparing BL-760 to ICG. Intravenous BL-760 identified three areas of bile leakage on the cut liver edge within 5 minutes (tumor-to-background ratio(TBR) 2.5-3.8), outperforming ICG, which was obscured by parenchymal signal and bleeding (**Figure 4D**). Repeated dosing confirmed leaks and revealed additional ones, highlighting its potential for real-time monitoring (141). These models mimic human anatomy, supporting translation to clinical use, though species differences in liver fat content may influence results. A milestone in the application of NIR−II technology to liver surgery was achieved in 2023 with the breakthrough first-in-human clinical trial. In this study, a visible-light/NIR-I/NIR-II multispectral imaging platform was developed and evaluated in a cohort of 23 patients with hepatocellular carcinoma (54). Intravenous indocyanine green ICG was administered preoperatively, and intraoperative NIR-I/II imaging enabled the detection of residual lesions that were challenging to identify using either direct visualization by surgeons or intraoperative ultrasonography, thereby facilitating timely surgical removal. Quantitative analysis revealed that NIR-II imaging provided superior image quality and a higher detection rate of malignant tumors compared with NIR-I imaging, with significantly elevated tumor-to-normal tissue (TNR) and TBR. Moreover, ICG-based intraoperative NIR-I/II fluorescence imaging detected tumor foci lacking conspicuous radiologic features, outperforming conventional preoperative imaging modalities ultrasonography, MRI, CT and intraoperative ultrasonography. Interestingly, unlike NIR-I imaging, which necessitates turning off operating room lights, NIR-II imaging is compatible with standard surgical illumination, offering enhanced practicality and feasibility for routine clinical implementation.

Pancreatic cancer surgery, whether performed via the Whipple procedure or distal pancreatectomy, remains challenging due to the low contrast between tumor tissue and the surrounding inflamed or fibrotic pancreas. This difficulty has driven growing interest in molecular imaging approaches designed to better highlight pancreatic tumors and metastatic lymph nodes during surgery. One avenue being explored is targeting pancreatic tumor biomarkers such as CEA or CA19–9 with fluorescent probes (142). In a recent preclinical study, a carcinoembryonic antigen–targeted nanobody conjugated to the NIR dye ZW800 was evaluated for pancreatic cancer, and in mouse models it demonstrated tumor-specific uptake that enabled intraoperative fluorescence imaging of pancreatic tumors (143). Although ZW800 emits in the high-700 nm range within the NIR-I window, the approach could be readily adapted by replacing it with a second-window dye to enhance imaging performance. According to the authors, NIR-II fluorophores with appropriate conjugation chemistry are currently in development for pancreatic cancer imaging. Another approach is integrating fluorescence with endoscopic surgery: for instance, a VEGF-A–targeted probe is currently in a Phase 1 trial for NIR-II fluorescence endoscopy in the GI tract (including pancreas) (144). The goal is to visualize premalignant or malignant lesions *in situ* during endoscopy with the enhanced contrast of NIR-II. For pancreatic tumors specifically, intraoperative NIR-II imaging might assist in margin assessment on the pancreatic transection surface or in detecting small metastatic nodules in the peritoneum or liver at the time of surgery, a scenario that often leads surgeons to abandon curative procedures (145). No clinical NIR-II probe is yet available for the pancreas, but ongoing research, such as minimally invasive nanoplatforms named HA/DOX AuNRs that are modified with hyaluronic acid and loaded with doxorubicin gold nanorings for pancreatic tumor-targeted photothermal chemotherapy, will eventually yield imaging agents (146, 147). A challenge in pancreas is that probes need to deeply penetrate the dense stroma; NIR-II’s deeper penetration could be beneficial here, potentially highlighting tumor in the pancreatic head even if the probe is administered systemically and has to perfuse through fibrosis (57). Hepatobiliary surgery has already made significant progress with the introduction of NIR-II guidance. Meanwhile, pancreatic surgery could benefit greatly once specific targeted probes—such as those against CA19-9, integrins, or the tumor stroma—are developed and applied in clinical practice.

Biliary surgery may also benefit: for instance, in gallbladder or bile duct cancer surgery, identifying bile ducts and margins is tricky. NIR-I with ICG is used to outline biliary anatomy, but NIR-II could improve how deep one can see bile ducts through liver tissue. There’s ongoing research on NIR-II cholangiography agents (148, 149).

#### 3.2.5 Colorectal and other gastrointestinal surgeries

Fluorescence-guided surgery for colorectal cancer has mostly relied on NIR-I techniques—for example, using ICG angiography to assess colon perfusion after resection or ICG mapping to identify lymph nodes (150). The second window is now being investigated to extend these capabilities. One promising application is peritoneal metastasis detection. Patients with colorectal cancer can develop tiny peritoneal implants that are hard to spot visually. NIR-II fluorescence may help pick up these small metastases under surgical illumination. Guo et al. recently demonstrated the utility of an organic NIR-II dye, which preferentially accumulates in tumors, for identifying peritoneal metastatic nodules in mice (92). Using NIR-II imaging, they detected nodules smaller than 1 mm that were missed by standard inspection. Furthermore, the study showed that NIR-II-guided resection of all fluorescent nodules significantly reduced tumor recurrence compared to unguided surgery. Clinically, this concept holds promise for applications in staging laparoscopy, where surgeons routinely perform an initial laparoscopy to assess for metastases prior to definitive colorectal cancer surgery. Administering a tumor-targeted NIR-II probe, either by spraying or injection, could therefore help detect hidden peritoneal disease more effectively.

A previous prospective cross-sectional study indicated that using ICG with an NIR-II camera to detect hidden peritoneal metastases in patients with colorectal or gastric cancer during diagnostic laparoscopy—by taking advantage of the near-infrared type II tail of ICG to enhance the visualization of serosal lesions—was both safe and highly sensitive (151). Early reports also noted that some sub-centimeter implants, invisible under white light, exhibited fluorescence and were therefore successfully identified (152).

For intraoperative margin assessment in colorectal surgery, the challenge is that most of the tumor is removed en bloc, and margins are checked by pathology later. However, for rectal cancer or anal cancer, where margins can be very close, a topical or injected NIR-II probe might help delineate the tumor border *in vivo*. For instance, Xu Z et al. demonstrated a NIR-II contrast agent that can be sprayed onto resected colon specimens to highlight microscopic tumor foci on the margin surfaces; if the detection results are positive, these foci are flagged for immediate re-excision. Furthermore, this spray enables near-instantaneous and accurate identification of tumor tissue, underscoring the ubiquity and versatility of near-infrared fluorescent sprays in clinical practice (153). In the future, one could envision an endoscopic delivery of a fluorescence probe along the rectal lumen to mark the distal margin of a rectal tumor before stapling, ensuring a sufficient clearance.

Another exciting development is molecular imaging of dysplastic polyps or early cancers during endoscopy. As noted, a VEGF-A–targeted NIR-II fluorescent peptide is under clinical trial in GI endoscopy (144). The goal is to improve detection of flat adenomas or Barrett’s dysplasia that can be missed in standard endoscopy. By attaching a NIR-II camera to the endoscope, the mucosa can be scanned for fluorescent “hotspots.” In theory, such an approach could increase polyp detection rates and cancer prevention. The CD24-targeted probe for colorectal neoplasia early detection also falls in this category – while still preclinical, it showed that even small (few mm) lesions in the colon could be visualized in second-window fluorescence (154). Given that NIR light can penetrate the superficial submucosa, NIR-II might even identify subsurface lesions that haven’t raised the mucosa yet.

In gastric cancer, NIR fluorescence can assist in detecting peritoneal metastases or sentinel lymph nodes—such as in the Japanese “Firefly” trial, which uses NIR for nodal mapping (155). In esophageal cancer, fluorescence imaging may help define surgical margins or reveal occult local spread (156). For these malignancies, targeted NIR-II probes directed against markers such as HER2 or specific mucins could further enhance surgical resection precision.

#### 3.2.6 Bone and soft tissue tumors

Surgical resection of bone and soft tissue tumors often requires a wide margin, and positive margins portend high local recurrence. However, taking wide margins in limbs can mean loss of function, so better intraoperative margin visualization is needed. Use of fluorescence in this field is still nascent. One difficulty is delivering probes into bone lesions; systemic administration works if the tumor has blood supply, but some bone tumors have poor perfusion (157). Nonetheless, preclinical studies provide a proof-of-concept. For example, in an orthotopic model of osteosarcoma, ICG was found to accumulate in the tumor and NIR imaging could outline the tumor boundary against normal bone owing to different perfusion, hinting that fluorescence might guide bone cuts in limb-sparing surgery (158). NIR-II probes could enhance this by deeper light penetration through cortical bone or surrounding muscle (159). A hypothetical targeted probe for osteosarcoma could be one against bone alkaline phosphatase or a bone matrix protein that is upregulated – labeled with a NIR-II dye to fluoresce at tumor sites (160). Likewise, for soft tissue sarcomas, which are often embedded in muscle planes, a second-window probe could help distinguish tumor from muscle intraoperatively, as muscle autofluorescence is low in the NIR-II region (161). While we did not find specific reported cases of NIR-II use in sarcoma surgery in the literature yet, it is reasonable to assume that general tumor-targeted probes, such as integrin-targeted NIR-II nanoparticles, could be applied. In one related study, the researchers used polyethylenimine(PEI) as a reducing agent and KMnO4 as a manganese source to efficiently synthesize mesoporous MnO_2_(mMnO_2_), which can be precisely controlled and coated onto the surface of UCNPs and other nanomaterials with different morphologies, resulting in composite materials with tunable functions. By integrating the photosensitizer Ce6, cisplatin, and tumor targeting pentapeptide (TFA), a therapeutic nanoplatform(named U6MDT) was developed, exhibiting tumor microenvironment (TME)-responsive properties and downconversion luminescence (DCL) in the NIR-II region. In a mouse model of bone metastasis, NIR-II imaging of DCL was employed to trace the accumulation of nanomaterials at the tumor site under 730 nm laser irradiation, and when combined with photodynamic therapy (PDT) and chemotherapy, the platform demonstrated excellent antitumor effects (162).We can reasonably predict that by combining drug carriers with technologies such as NIR-II and PDT, composite probes can provide multiple functions in treatment, and this efficient integrated platform holds great potential as an important reference for the precise treatment of metastatic bone tumors.

Another area of interest involves melanoma and soft-tissue skin tumors. Melanoma resections are sometimes guided by visible dyes, such as isosulfan blue for node detection, and by emerging Raman or mass spectrometry imaging tools (163–165). NIR-II fluorescence could prove particularly valuable in this setting, since melanin absorbs near-infrared light—although it primarily acts as a quencher (166). A melanocyte-targeted NIR-II probe, for instance one directed against MC1R or based on a scaffold that binds melanin, might enable surgeons to visualize the true margins of a melanoma beyond visibly pigmented areas (167). Similarly, in abdominal sarcomas such as retroperitoneal sarcomas, NIR-II imaging could help confirm complete tumor removal, as it penetrates fat more effectively and scatters less than shorter wavelengths (168). Although practical applications in bone and soft-tissue tumor surgery remain limited, the potential benefits are clear. The key obstacle at present is the lack of tumor-specific probes for these rare cancers, which remains an unmet need that future research should aim to address.

#### 3.2.7 Reconstructive and vascular surgery

In reconstructive surgery-such as free flap transfers, replantation of digits, or lymphatic surgery-surgeons need to assess blood flow through small vessels and map lymphatic channels. Traditionally, vascular assessment has relied mainly on clinical observation methods such as the capillary refill test, or on Doppler ultrasonography to evaluate vascular conditions, as well as the use of blue dye lymphangiography to visualize the lymphatic system (169). With the advent of near-infrared fluorescence with ICG, this field has undergone significant transformation. ICG angiography can now be used to assess flap perfusion and the patency of vascular anastomoses: after intravenous injection of the dye, its flow path within the flap vasculature can be observed in real time under a near-infrared camera (170). However, most current systems visualize ICG in the NIR-I window. By moving to NIR-II detection, one can gain better sensitivity and penetration (**Figure 5**). An exploratory, pioneering clinical study conducted in Wuhan, China, evaluated a portable NIR-II imaging system in conjunction with ICG in a cohort of 39 patients undergoing microsurgical procedures, including digital replantation and flap transplantation (89). They found that NIR-II imaging could clearly visualize blood flow in both arteries and veins of the surgical site after anastomosis, and importantly, the images were much cleaner than the simultaneous NIR-I images. Under NIR-I, they noted more background autofluorescence and “fuzzy” delineation of vessels, especially at longer post-injection times, as the fluorescence in surrounding tissues increased. In contrast, NIR-II images showed the vessels with high contrast and minimal background, even minutes after ICG injection This allowed more accurate and faster judgment of whether a reconnected artery/vein was open or if there was a blockage.

**Figure 5:**
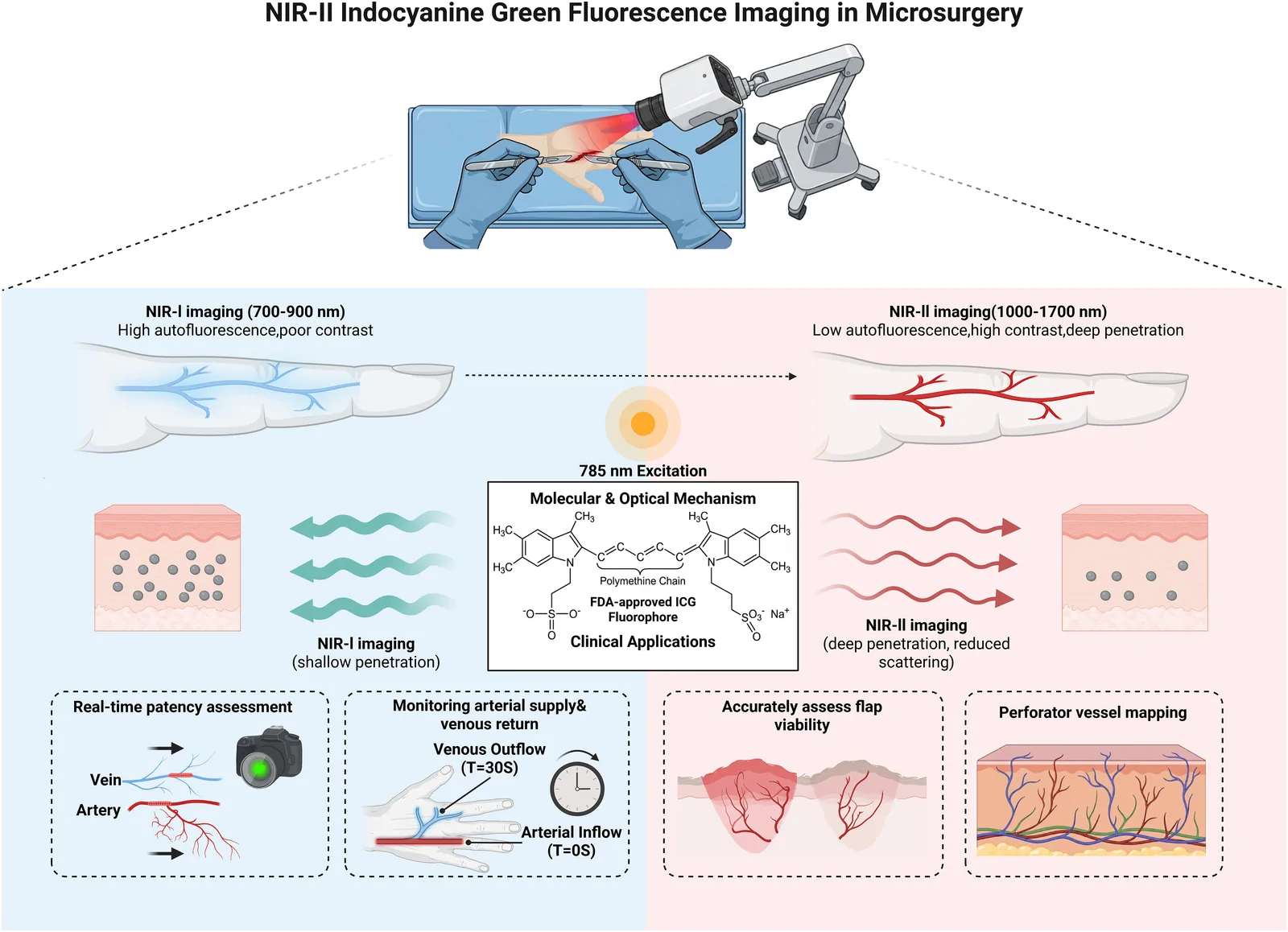
NIR-ll indocyanine green fluorescence imaging in microsurgery. NIR-I imaging: Vascular margins appear blurred with significant tissue autofluorescence and a low SBR; NIR-II imaging: Vasculature is delineated with sharp, crisp contours, high contrast, and minimal background fluorescence. Schematic representation of photon-tissue interactions for ICG under 785 nm laser excitation. NIR-I window: ICG emits shorter, broader wavelengths that interact with abundant scattering particles within the tissue, resulting in shallow tissue penetration. NIR-II window: ICG emits longer, narrower wavelengths that interact with fewer scattering particles, demonstrating enhanced penetration depth. Clinical applications of NIR-II imaging. Real-time vascular patency assessment: Following successful arteriovenous anastomosis, NIR-II imaging clearly visualizes blood flow. Rapid fluorescence onset indicates arterial inflow, followed by a slow washout phase representing venous drainage. Perfusion mapping in flap surgery: NIR-II imaging reveals the flap with a distinct vascular network, differentiating well-perfused areas from potentially ischemic regions.

Similarly, in lymphatic surgery, ICG lymphography is common to map lymphatic channels (171). The swine study by Wang et al. demonstrated that ICG-based NIR-II imaging could dynamically track lymph flow and identify lymph leakage or obstruction sites better than NIR-I (53). Under NIR-II imaging, researchers were able to visualize lymphatic vessels located deep beneath the skin and subcutaneous fat layers, which are often indiscernible in NIR-I imaging. These findings suggest that NIR-II imaging enables more effective discrimination between functional and dysfunctional lymphatics in patients with lymphedema, a population often characterized by tissue thickening and fibrosis that complicates diagnosis. In fact, the first-in-human microsurgery study also explored perforator flap planning. Before surgery, the team used NIR-II ICG imaging to identify skin perforator vessels for flap harvesting. This approach revealed a significantly higher number of perforators and provided a much clearer visualization of their vascular territories compared with traditional handheld Doppler ultrasound mapping. In all nine flap patients, NIR-II identified perforators that Doppler missed, and those vessels were confirmed during surgery. Moreover, NIR-I imaging of the same area was less clear and suffered from autofluorescence over time, whereas NIR-II gave a crisp picture of each perforator’s location and flow. All flaps survived, indicating that using NIR-II to choose well-perfused tissue can improve outcomes.

Building on these successes, the use of NIR-II imaging in non-tumor surgery is likely to grow. Its adoption could extend to vascular procedures, for example, allowing surgeons to assess limb bypass graft perfusion during surgery or to visualize blood flow in real time during endovascular interventions (172, 173). In transplantation, NIR-II could help evaluate organ perfusion directly *in situ*. After a liver transplant, for instance, surgeons might inject ICG and use NIR-II imaging to confirm that the hepatic artery and portal vein are evenly perfusing the liver—an assessment that currently depends on Doppler ultrasound and clinical judgment (174). Thanks to its greater depth of tissue penetration, NIR-II may even make it possible to observe perfusion in deeper organs during open surgery without bringing the camera too close or needing to exteriorize the organ as much. Applications in related fields based on this advantage are illustrated in **Figure 6**.

**Figure 6:**
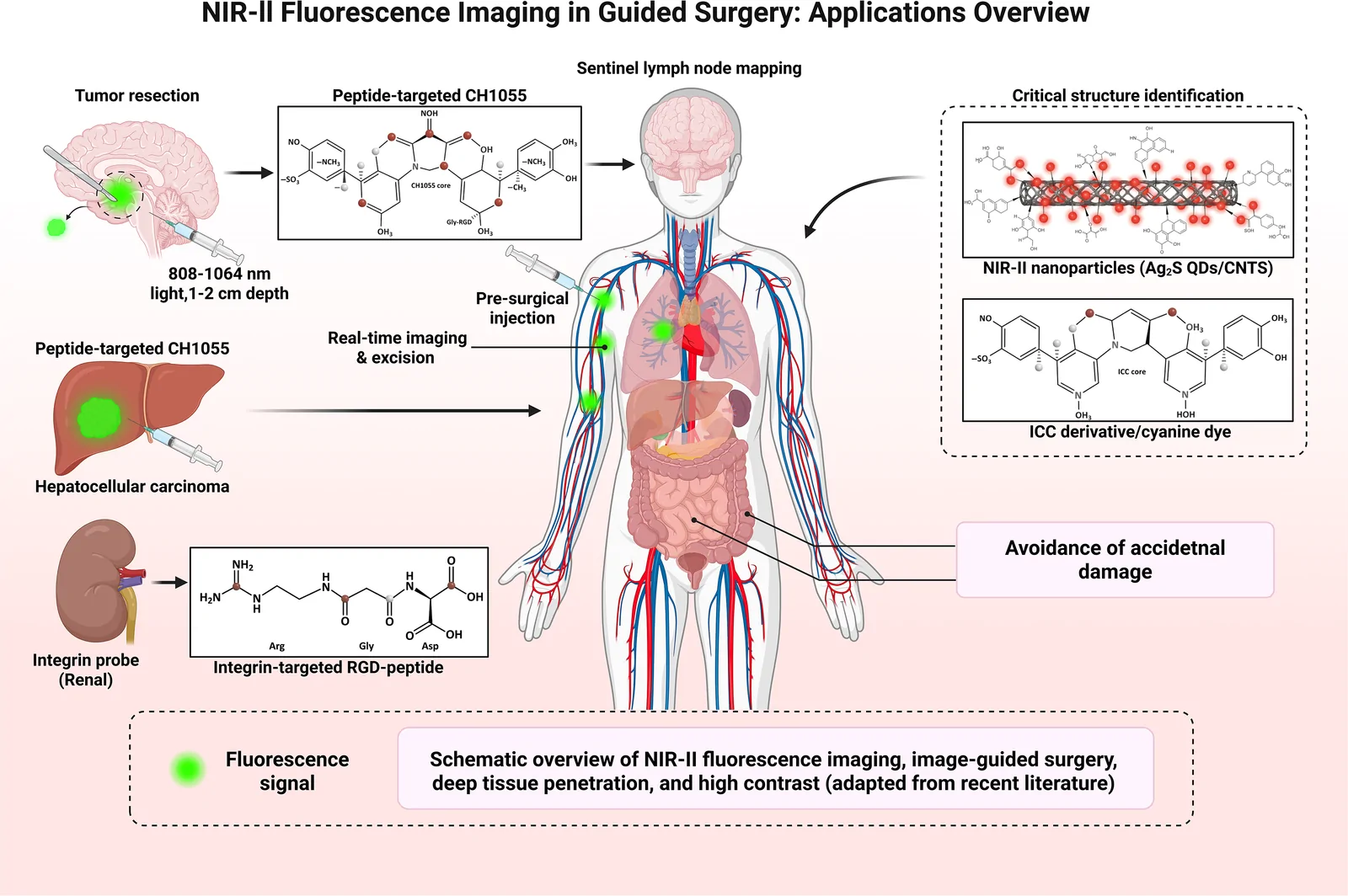
Overview of NIR-II fluorescence imaging applications in guided surgery. Tumor resection in multiple organs is performed using targeted NIR-II probes, such as the peptide-targeted dye CH1055 for gliomas and integrin-targeted probes for renal tumors, to achieve clear tumor margins. Preoperative intravenous injection combined with real-time intraoperative imaging guidance enables NIR-II imaging to support sentinel lymph node mapping. NIR-II nanoparticles and ICC-derived cyanine dyes provide clear identification of critical anatomical structures, such as blood vessels and nerves, thereby reducing the risk of accidental injury.

Finally, it’s worth noting that NIR-II imaging can often be done under normal ambient light, which is very advantageous in busy surgical fields (175). In the microsurgery study, the NIR-II system was used in the OR without turning off the lights, and thanks to filtering and time-gated imaging, they still obtained clear images (53). This makes it practical for surgeons to incorporate fluorescence imaging intermittently during a procedure without workflow disruption.

### 3.3 Clinical translation: current trials and future challenges

Despite the rapid development of near-infrared-II (NIR-II) fluorescent probes, their clinical translation remains constrained by regulatory barriers. At the current stage, repurposing clinically established NIR-I fluorophores with detectable shortwave-infrared (SWIR) tail emission represents a practical near-term translational strategy. Carr et al. demonstrated that ICG retains measurable emission beyond 1000 nm and enables SWIR imaging in preclinical models (71). This finding suggests that, when combined with appropriate SWIR-sensitive detection systems, NIR-I fluorophores may facilitate the clinical translation of NIR-II imaging.

However, SWIR imaging based on tail emission should be regarded as a transitional strategy rather than an ideal ultimate solution. Because the detected signal originates from the relatively weak long-wavelength emission tail rather than peak emission within the NIR-II window, its imaging performance may be limited by photon efficiency, tissue background, the fluorophore microenvironment, and imaging instrumentation. This limitation was highlighted by Keizers et al., who compared NIR and SWIR imaging in clinical tumor specimens containing cetuximab-IRDye800CW (123). Although SWIR emission was detectable, SWIR imaging did not consistently outperform conventional NIR imaging. It showed comparable performance to NIR imaging in penile squamous cell carcinoma samples, whereas in head and neck squamous cell carcinoma samples, SWIR imaging performed worse across several evaluation metrics. Therefore, repurposing NIR-I fluorophores through SWIR tail emission is a clinically realistic but highly context-dependent strategy. From a clinical perspective, close collaboration with multidisciplinary experts is essential to develop NIR-II probes with higher brightness, favorable pharmacokinetic properties, and reliable safety profiles, which will be critical for advancing fluorescence-guided surgery in the future.

#### 3.3.1 Completed and ongoing clinical studies

A five-year follow-up clinical study was designed to evaluate whether patients undergoing NIR-II-guided hepatectomy for HCC exhibited prolonged recurrence-free survival (RFS) (176). The results demonstrated that the more radical resections achieved via fluorescence guidance did indeed extend RFS in HCC patients, accompanied by a lower recurrence rate. These findings provide robust clinical evidence supporting the broad application of NIR-II imaging in oncologic surgery.

In the field of oncologic surgery trails: -A phase I clinical trial in China evaluated a novel NIR-II dye, known as IR-FD, in patients with gastrointestinal cancers to visualize tumor fluorescence. The findings have so far been reported in conference abstracts and have not yet been fully published (177). They reported that the dye helped identify tumors during surgery similarly to how ICG was used, but with potentially better tumor specificity. The VEGF-A targeted endoscopy trial is recruiting as mentioned (144). If it shows that NIR-II can find polyps or early lesions better, it could open a new paradigm in GI oncology for early intervention. SGM-101, a CEA-targeted NIR-I probe, though not belonging to the NIR-II class, recently completed a Phase III clinical trial in colorectal cancer surgery with promising outcomes, enhancing the detection of lesions that were otherwise difficult to visualize (178). This success paves the way for similar targeted approaches in NIR-II. In fact, one could envision a next-gen SGM-101 using a NIR-II dye to allow deeper detection of tumor deposits in mesentery or lymph nodes. The underlying principle remains the same-specific tumor labeling via antibodies-yet the utilization of NIR-II imaging enables the detection of tumor clusters located at greater depths or those previously deemed elusive. 

Pafolacianine (OTL38; Cytalux), a folate receptor-targeted near-infrared optical imaging agent, received FDA approval in November 2021 as an adjunct for the intraoperative identification of malignant lesions in adult patients with ovarian cancer (179). Its clinical efficacy was evaluated in a phase III, open-label, multicenter trial, in which 150 patients received intravenous pafolacianine. In this study, pafolacianine-guided near-infrared imaging identified additional cancer tissue that had not been planned for resection and was not detected by white-light assessment or palpation in 33.0% of patients, exceeding the prespecified threshold of 10%; among patients undergoing interval debulking surgery, the detection rate reached 39.7% (180). The development of a NIR-II folate probe could potentially enable more effective penetration through ascites or fibrotic adhesions, thereby allowing for more complete cytoreductive surgery.

At present, there are still no NIR-II–specific probes that have received full regulatory approval for clinical marketing, apart from the occasional repurposing of ICG itself (71). The first to cross that threshold may come from a few promising fronts. One possibility lies in the improved cyanine dyes—ICG derivatives such as ICG-C9—which, given their chemical similarity to ICG, could benefit from a more straightforward safety evaluation (181). Another strong contender could be certain polymer dots, as some have demonstrated no measurable toxicity in animal studies and exhibit simple, predictable excretion pathways (182). Meanwhile, more complex targeted probes will likely advance through the formal investigational new drug (IND)route; for instance, an antibody–NIR-II dye conjugate may soon reach the clinical trial stage in either the U.S. or the EU.

A critical ongoing effort is to validate that using these probes truly changes surgical decision-making and patient outcomes. For instance, in breast cancer surgery, trials are being designed to see if fluorescence can reduce re-excision rates (183). If NIR-II probes enable clearer delineation of tumor margins, they may further reduce the positive margin rate. Consequently, clinical trials should prioritize endpoints such as the detection of additional lesions identified by fluorescence labeling and differences in local recurrence. Representative clinical studies and trials involving fluorescence-guided surgery are summarized in **Table 4** to illustrate the current status of translational medicine research.

**Table 4:** Selected clinical studies and trials of FGS involving NIR-II imaging or related molecular probes.

Study/trial	Indication/procedure	Fluorophore & window	Key findings/status	Refs
First-in-human NIR-II liver tumor imaging	Liver cancer resection (open surgery)	ICG (emission tail in NIR-II)	NIR-II imaging detected 100% of tumors vs 90.6% with NIR-I. Identified residual tumor intraoperatively that was missed by surgeon/ultrasound. No adverse events; demonstrated feasibility of dual NIR-I/NIR-II system.	(54)
Microsurgery vascular assessment	Reconstructive microsurgery (e.g., finger replantation, flap surgery)	ICG (NIR-II imaging device)	39 patients: NIR-II imaging clearly visualized blood flow in tiny vessels and flaps. Enhanced identification of perforators and anastomoses, improving surgical accuracy. Showed NIR-II’s safety and utility beyond oncology.	(89)
CEA-targeted FGS in colon cancer (Phase II, 2018) – SGM-101 trial, Netherlands	Colorectal cancer surgery (open resection)	SGM-101 (CEA antibody + 700 nm dye, NIR-I)	SGM-101 detected additional tumor lesions intraoperatively in 34% of patients, influencing surgical strategy. Improved R0 resection rates reported. (NIR-I result showing value of targeting; NIR-II analogs anticipated.)	(178)
VEGF-A targeted NIR-II endoscopy (initiated 2024)	Gastrointestinal polyp/cancer detection (endoscopy)	Novel anti-VEGF probe with NIR-II endoscope	Ongoing: Aims to demonstrate feasibility of NIR-II endoscopic imaging. Outcomes: ability to highlight abnormal GI mucosa and safety of probe. Could set stage for NIR-II in early cancer diagnosis.	(144)
HDAC6-targeted HCC imaging	Hepatocellular carcinoma (open surgery, plus ex vivo)	SeCF_3_-IRDye800 (small mol. target, NIR-I/NIR-II)	Preclinical/Translational: Intraoperative NIR-II imaging in an HCC model improved detection of residual tumor nodules vs ICG. Also showed probe binding to human tumor specimens. Positions this targeted probe for first-in-human trial in liver cancer.	(95)
Glioma surgery with 5-ALA	Malignant glioma resection (neurosurgery)	5-ALA (Visible fluorescence)	Phase III showed 5-ALA FGS increased complete resection to 65% vs 36% in controls, improving 6-month PFS.	(18)
Multi-center trial of Panitumumab-IRDye800 (ongoing)	Head & Neck cancer surgery	Panitumumab (anti-EGFR) + IRDye800 (NIR-I)	Phase II ongoing in US: thus far showed ability to detect microscopic residual in surgical cavity and positive margins that were not apparent otherwise. Expected to report on sensitivity/specificity of margin detection.	(125)

#### 3.3.2 Challenges

Regulatory Approval of New Probes: Bringing a new fluorescent probe to the clinic is much like introducing a new drug, as it requires extensive toxicity testing, dose optimization, and clinical validation. Many NIR-II dyes and nanoparticles are entirely new chemical structures, and their long-term safety remains uncertain (110). Regulators will expect thorough evaluation of these compounds before approval. The process is somewhat faster if the new probe builds on existing materials, such as ICG analogs or approved antibodies linked with a different dye (184). Still, as Rosenthal and colleagues have noted, the time and cost involved in getting both a new imaging agent and its device approved can discourage industry participation (19). One possible solution is to repurpose existing, approved imaging systems for use with new probes, or vice versa, which could shorten the path to the clinic.

Device Availability: For NIR-II imaging to be widely adopted, the technology must be accessible to surgeons. Right now, only a handful of research hospitals are equipped with InGaAs cameras capable of detecting NIR-II fluorescence (81). What’s needed are compact, affordable, and easy-to-use systems, ideally as add-ons for surgical microscopes or laparoscopes. Encouragingly, several companies are already pursuing this endeavor, developing NIR-II-capable systems inspired by successful NIR-I devices such as Fluobeam and VisionSense (185). As technology continues to improve, we can expect better image quality, lower costs, and broader clinical availability.

Training and Interpretation: As with any new imaging technology, surgeon training is vital. The fluorescence view provided by NIR-II imaging is very different from what surgeons are used to seeing. A bright fluorescent signal might prompt additional resection or biopsy, so it’s crucial that clinicians understand what the signal really means (186). False positives from inflammation or benign tissues can occur, and without proper interpretation, this could lead to overtreatment. In head and neck trials, for instance, surgeons have to learn that sometimes salivary glands or inflammation can cause nonspecific fluorescence (187). Experiences from early clinical trials show that education and hands-on familiarity are essential for accurate use and good surgical decision-making.

Cost-effectiveness: Finally, hospitals will need to weigh the financial investment in new imaging systems and probes against their clinical benefits. Demonstrating cost-effectiveness, for example by reducing reoperations or improving surgical outcomes, will be key to encouraging adoption. Health economics studies following early clinical trials will help determine this balance. In many cases, preventing even one repeat surgery or avoiding the need to convert to an open procedure could justify the added expense (188).

## 4 Conclusion and future perspectives

NIR-II fluorescence imaging is revolutionizing the field of precision surgery, and it is imperative for surgeons to fully leverage this technology. In fact, our center is currently utilizing ICG for intraoperative guidance during osteosarcoma resections to better define surgical margins and identify residual tumor tissue. Concurrently, we are developing novel NIR-I and NIR-II probes, with the aim of advancing them to clinical validation in the near future. Unlike conventional methods that are often time-consuming and prone to missing microscopic lesions, NIR-II molecular probes generate high-resolution, high-contrast images and enable real-time navigation. These capabilities address critical challenges in surgical oncology: achieving R0 resection by delineating residual tumor tissue, while precisely outlining the target area to preserve healthy tissue. Consequently, this remains a primary focus of our research.

Looking ahead, the application prospects of NIR-II imaging in surgery are highly promising. Several key directions of future development can be anticipated. First, the design of more intelligent probes involves multifunctional agents that are capable not only of tumor visualization but also of therapeutic drug delivery, known as theranostic probes, or agents that simultaneously emit multiple imaging signals, such as fluorescence and photoacoustic signals, which may enable deeper and more comprehensive visualization. Second, exploration of the NIR-III window (1700–2000 nm): longer wavelengths may further enhance imaging depth. Although detectors and probes for this spectral range remain at an early stage of development, wavelengths beyond 1500 nm have already been categorized as NIR-IIb or NIR-III in recent studies (189, 190). Researchers are actively working to overcome technical barriers and achieve whole-organ imaging in small animal models. Third, integration with other imaging technologies: for example, combining fluorescence imaging with AR could allow real-time overlay of NIR-II fluorescence signals directly onto the surgeon’s field of view. While overlay projection has already been implemented for the NIR-I window, achieving this in the NIR-II window requires more precise calibration and advanced software support; nevertheless, it is technically feasible (191). Finally, broader clinical indications beyond oncology are expected. For instance, in spinal or otolaryngological surgery, myelin-binding NIR-II probes could be used to identify neural structures and thus reduce the risk of nerve injury (192). In transplantation surgery, NIR-II imaging may facilitate the detection of ischemic tissues to ensure adequate perfusion (193). Applications in microsurgery have already demonstrated its value in vascularized flap procedures (89).

Within just over a decade, NIR-II molecular imaging has progressed from a conceptual framework to early clinical implementation. This technology offers surgeons a significant advantage by clearly delineating lesion margins that are invisible to the naked eye, directly addressing one of the most critical challenges in surgical oncology: achieving complete tumor resection while preserving as much healthy tissue as possible. Recent studies and early clinical trials have demonstrated that NIR-II imaging provides sharper lesion boundaries, enables detection of deeper pathologies, and improves surgical precision (194). Although challenges remain in regulatory approval, safety evaluation, and technical integration, progress to date has been rapid and steady. With continued collaboration among chemists, engineers, and surgeons, the vision of truly molecular-level precision-guided surgery is rapidly becoming a reality.
